# Theoretical Considerations Regarding the Role of Hyperinsulinemia and Insulin/IGF Signaling in Cancer Metabolic Reprogramming: Potential Mechanisms and Therapeutic Vulnerabilities

**DOI:** 10.3390/ijms27219598

**Published:** 2026-10-27

**Authors:** Byeong Suk Chae

**Affiliations:** College of Pharmacy, Woosuk University, Wanju 55338, Republic of Korea; cbse@woosuk.ac.kr

**Keywords:** hyperinsulinemia, insulin/IGF signaling, metabolic reprogramming, metabolic plasticity, PI3K–AKT–mTOR signaling, tumor microenvironment

## Abstract

Hyperinsulinemia is increasingly recognized as a systemic factor that can promote tumor progression by amplifying insulin receptor and insulin-like growth factor (IGF) signaling, reshaping cancer-cell metabolism, and influencing the tumor microenvironment. Commonly associated with obesity, type 2 diabetes, and metabolic syndrome, persistent hyperinsulinemia can enhance insulin/IGF receptor signaling and downstream PI3K–AKT–mTOR and RAS–MAPK pathways. These pathways interact with stress-response networks, including NRF2-dependent redox control and AMPK–autophagy signaling, thereby supporting metabolic plasticity under nutrient and microenvironmental stress. Hyperinsulinemia-associated signaling may increase glucose utilization, pentose phosphate pathway activity, glutaminolysis, de novo lipogenesis, and antioxidant capacity, collectively promoting anabolic growth, survival, and treatment adaptation. Metabolic remodeling extends beyond aerobic glycolysis and involves context-dependent alterations in mitochondrial function, oxidative metabolism, lactate production, and metabolic crosstalk among cancer, stromal, and immune cells. Consequently, hyperinsulinemia-associated signaling may contribute to lactate-rich, immunosuppressive tumor microenvironments, tumor progression, and heterogeneous therapeutic responses across cancer types. This review integrates experimental, epidemiological, and translational evidence linking persistent hyperinsulinemia and insulin/IGF signaling with cancer metabolic reprogramming. It critically examines strategies to reduce systemic insulin exposure, inhibit insulin/IGF signaling, or co-target tumor-specific metabolic dependencies, including the metabolic consequences of PI3K-pathway inhibition. By advancing a hyperinsulinemia-centered framework, this review identifies mechanistic gaps and translational priorities for more precise metabolic interventions in cancer.

## 1. Introduction

Cancer is characterized by sustained proliferative signaling, resistance to cell death, and metabolic reprogramming that reshapes glucose, lipid, and amino acid utilization. To sustain growth and survival within a hostile tumor microenvironment (TME) characterized by oxidative stress, nutrient limitation, hypoxia, and therapeutic pressure, cancer cells undergo dynamic metabolic remodeling that supports energy production, redox homeostasis, and the generation of biosynthetic precursors required for proliferation, invasion, and metastasis [1,2,3,4]. A central feature of this remodeling is aerobic glycolysis, commonly known as the Warburg effect, in which cancer cells maintain high glucose uptake and lactate production despite oxygen availability. This metabolic state supports rapid glycolytic flux and the diversion of carbon intermediates toward nucleotide, amino acid, and lipid biosynthesis [5,6]. In parallel, de novo lipogenesis, glutaminolysis, and the pentose phosphate pathway (PPP) provide anabolic substrates and reducing equivalents, including nicotinamide adenine dinucleotide phosphate (NADPH), thereby increasing tumor adaptability under metabolic and oxidative stress [6,7].

Hyperinsulinemia is a common feature of insulin-resistant states associated with obesity, type 2 diabetes, and metabolic syndrome. It has been associated with increased cancer risk and adverse outcomes in several malignancies, although the magnitude and direction of these associations vary across tumor types and clinical contexts [8,9,10,11]. Epidemiological, experimental, and translational evidence suggests that sustained insulin and insulin-like growth factor (IGF) signaling, together with hyperglycemia, obesity-associated chronic low-grade inflammation, altered adipokine signaling, and impaired immune surveillance, may create a protumorigenic systemic environment. These host-derived factors can interact with tumor-intrinsic oncogenic alterations to promote metabolic remodeling and tumor progression [9,12,13].

At the cellular level, insulin is a major anabolic hormone that regulates glucose uptake, glycolysis, lipid biosynthesis, and protein translation [14]. In tumor cells, signaling through the insulin receptor (IR), insulin receptor isoform A (IR-A), insulin-like growth factor 1 receptor (IGF-1R), and IR/IGF-1R hybrid receptors can activate the phosphoinositide 3-kinase–AKT–mechanistic target of rapamycin (PI3K–AKT–mTOR) and RAS–mitogen-activated protein kinase (RAS–MAPK) cascades [12,15,16,17]. These pathways integrate growth-factor, nutrient, and stress signals and can support proliferation, survival, metabolic plasticity, and, in selected settings, therapeutic resistance.

During hyperinsulinemia, increased systemic insulin exposure, together with context-dependent changes in IGF ligand bioavailability, may amplify signaling through IR, IGF-1R, and hybrid receptors. In susceptible tumors, receptor overexpression, altered expression of IR isoforms, and downstream oncogenic alterations may further sustain this signaling [15,16]. Such signaling can enhance glucose uptake, glycolytic flux, and lipogenesis while remodeling redox homeostasis through context-dependent effects on reactive oxygen species (ROS) production, antioxidant defenses, and genomic stress [18,19]. In selected tumor contexts, NRF2-dependent transcriptional programs can further support antioxidant capacity and anabolic metabolism by redirecting glucose and glutamine toward NADPH-generating pathways [20,21]. Hyperinsulinemia may therefore act as a systemic metabolic modifier that reinforces oncogenic signaling networks and favors glycolytic, anabolic, and lipogenic programs in susceptible tumor contexts.

Persistent insulin/IGF signaling can also cooperate with oncogenic mutations and tumor-suppressor loss to amplify metabolic remodeling, malignant transformation, and treatment adaptation [12,18]. This interaction creates potential therapeutic opportunities, including strategies that reduce systemic insulin exposure, inhibit insulin/IGF signaling, target the PI3K–AKT–mTOR or RAS–MAPK pathways, and exploit tumor-specific metabolic dependencies. Such approaches may suppress tumor growth and, in selected settings, enhance responsiveness to anticancer therapies; however, compensatory endocrine and metabolic feedback, tumor heterogeneity, and host metabolic status can limit therapeutic efficacy [9,22,23].

Previous reviews have comprehensively examined the relationships among obesity, diabetes, hyperinsulinemia, insulin/IGF signaling, and cancer, providing important foundations for understanding cancer risk, progression, and therapeutic responses [9,12,24]. Building on these syntheses, this review critically examines how persistent hyperinsulinemia may influence glycolytic, pentose phosphate, lipid, glutamine, mitochondrial, and redox metabolism through interactions among systemic insulin exposure, receptor-level signaling, and tumor-intrinsic metabolic plasticity. Particular emphasis is placed on PI3K–AKT–mTOR and RAS–MAPK signaling, NRF2/ROS-associated redox regulation, and AMPK–autophagy networks, with attention to tumor–host metabolic crosstalk and treatment-induced endocrine feedback.

Therapeutic strategies targeting systemic insulin exposure, insulin/IGF signaling, and tumor-specific metabolic vulnerabilities are evaluated in relation to context dependence, resistance mechanisms, and translational limitations. To strengthen the experimental orientation of the review, unresolved mechanistic questions are organized into three testable hypotheses concerning direct metabolic effects of elevated insulin, adaptation to nutrient stress, and molecular predictors of insulin-dependent metabolic responses. For each hypothesis, proposed experimental approaches, functional endpoints, and outcomes that would challenge or refute the proposed mechanism are specified. The intended contribution is a structured, experimentally oriented research framework that distinguishes established findings from testable propositions and guides future investigation.

## 2. Hyperinsulinemia, Insulin Resistance, and Cancer: Mechanistic Links and Metabolic Consequences

### 2.1. Hyperinsulinemia and Insulin Resistance: Bidirectional Metabolic Dysregulation

Hyperinsulinemia, defined as persistently elevated circulating insulin concentrations, commonly accompanies insulin-resistant states associated with obesity, type 2 diabetes, and metabolic syndrome [8,25,26]. Although compensatory hyperinsulinemia is classically regarded as a response to insulin resistance, experimental and longitudinal evidence suggests that chronic hyperinsulinemia may precede and contribute to insulin resistance in selected metabolic contexts [25,27]. The direction and magnitude of this relationship vary across tissues, disease stages, genetic backgrounds, and environmental exposures.

Chronic energy surplus, ectopic lipid deposition, inflammatory stress, and impaired hepatic insulin clearance can each contribute to elevated circulating insulin concentrations and reduced insulin sensitivity [25,26,27,28]. Conversely, sustained insulin exposure may promote receptor and post-receptor desensitization in metabolic tissues, whereas insulin resistance can stimulate compensatory insulin secretion to maintain glucose homeostasis [26]. These processes can create a self-reinforcing cycle involving hepatic steatosis, skeletal-muscle lipid accumulation, endoplasmic reticulum stress, mitochondrial dysfunction, and chronic low-grade inflammation.

These systemic disturbances are relevant to cancer because they can increase exposure to insulin and IGF-related mitogenic signals while coexisting with hyperglycemia, altered lipid availability, inflammatory cytokines, and oxidative stress [29,30]. However, these factors should not be considered uniform or interchangeable drivers, because their relative contributions to tumor initiation, progression, and therapeutic response are likely to be tumor- and context-dependent. Among these interconnected abnormalities, persistent hyperinsulinemia is particularly relevant because it can directly increase systemic exposure to growth-promoting insulin/IGF receptor signaling.

### 2.2. Linking Hyperinsulinemia to Cancer: Epidemiological Associations and Biological Mechanisms

Hyperinsulinemia and insulin resistance have been associated with increased risk and adverse outcomes in several malignancies, including endometrial, colorectal, liver, pancreatic, breast, ovarian, and prostate cancers [8,9,10,11]. However, the magnitude and direction of these associations vary according to tumor type, menopausal or hormonal status, metabolic phenotype, adiposity, and treatment context. Associations have also been reported in some non-obese populations, indicating that metabolic dysfunction may confer cancer-relevant risks that are not fully captured by body mass index alone [11,31]. Epidemiological evidence supports an association between insulin-resistant states and cancer; however, distinguishing the independent contribution of hyperinsulinemia from obesity, hyperglycemia, inflammation, altered sex-steroid signaling, and other metabolic disturbances remains challenging.

Several interacting mechanisms may link hyperinsulinemic and insulin-resistant states to tumorigenesis and tumor progression. Hyperglycemia, chronic inflammation, dysregulated adipokine signaling, oxidative stress, impaired antitumor immune surveillance, and increased bioavailability of IGF ligands may collectively generate a systemic milieu permissive for malignant growth [29,30]. Hyperinsulinemia may reduce hepatic production of sex hormone-binding globulin (SHBG), thereby increasing the circulating bioavailability of sex steroids. Together with obesity-related changes in sex-steroid metabolism, this mechanism may contribute to hormone-sensitive tumor biology, particularly in endometrial cancer and selected breast cancer contexts [32].

Insulin-resistant states may contribute to tumor microenvironment remodeling through altered nutrient availability, systemic inflammation, adipokine dysregulation, and metabolic interactions between tumor and stromal cells [29,33]. Lactate-associated metabolic crosstalk can further influence stromal activation, extracellular acidification, immune-cell function, and tumor progression in selected contexts [34,35]. The contribution of adipocyte- or stromal cell-derived lactate to tumor-niche remodeling should, however, be regarded as a plausible and context-dependent mechanism rather than a universal feature of hyperinsulinemia-associated cancer. Collectively, these findings support a biologically plausible contributory role for hyperinsulinemia in cancer development and progression while emphasizing substantial tumor-specific and patient-specific heterogeneity. Interventions that reduce systemic insulin exposure or target insulin/IGF-associated signaling have shown activity in experimental settings, but their clinical utility and effects on cancer outcomes require rigorous prospective evaluation.

The molecular basis of this context dependence is partly determined by receptor composition, ligand availability, downstream oncogenic alterations, and the metabolic state of both tumor and host tissues.

## 3. Key Mechanisms Linking Insulin Signaling to Cancer Promotion

### 3.1. IR-A and IGF Receptor Overexpression as Drivers of Oncogenic Signaling

The insulin receptor (IR) and insulin-like growth factor (IGF) signaling networks are dysregulated in many cancers and can support proliferation, survival, metabolic adaptation, and therapeutic resistance. IR and IGF-1R can form hybrid receptors composed of one IR half-receptor and one IGF-1R half-receptor. Their ligand-binding properties and downstream outputs depend partly on the incorporated IR isoform and the local availability of insulin, IGF-I, and IGF-II, thereby expanding the range of tumor-cell responses to endocrine and paracrine ligands [15,16]. In tumors expressing IR, IGF-1R, or IR/IGF-1R hybrid receptors, elevated circulating insulin and locally produced IGF ligands may amplify mitogenic and metabolic signaling; the magnitude of this effect is influenced by receptor abundance, ligand availability, receptor isoform composition, and downstream oncogenic alterations [16,17].

IR-A, the exon 11-negative IR isoform, binds insulin and IGF-II and has a substantially higher affinity for IGF-II than IR-B. It is relatively enriched in fetal tissues and in several malignancies, where it can increase responsiveness to both systemic insulin and locally available IGF-II [15,16,36]. In cancer, co-expression of IGF-II, IR-A, IGF-1R, and IR/IGF-1R hybrid receptors can establish autocrine and paracrine signaling circuits that may support tumor growth, survival, invasion, and therapeutic resistance [16,36].

**Figure 1:**
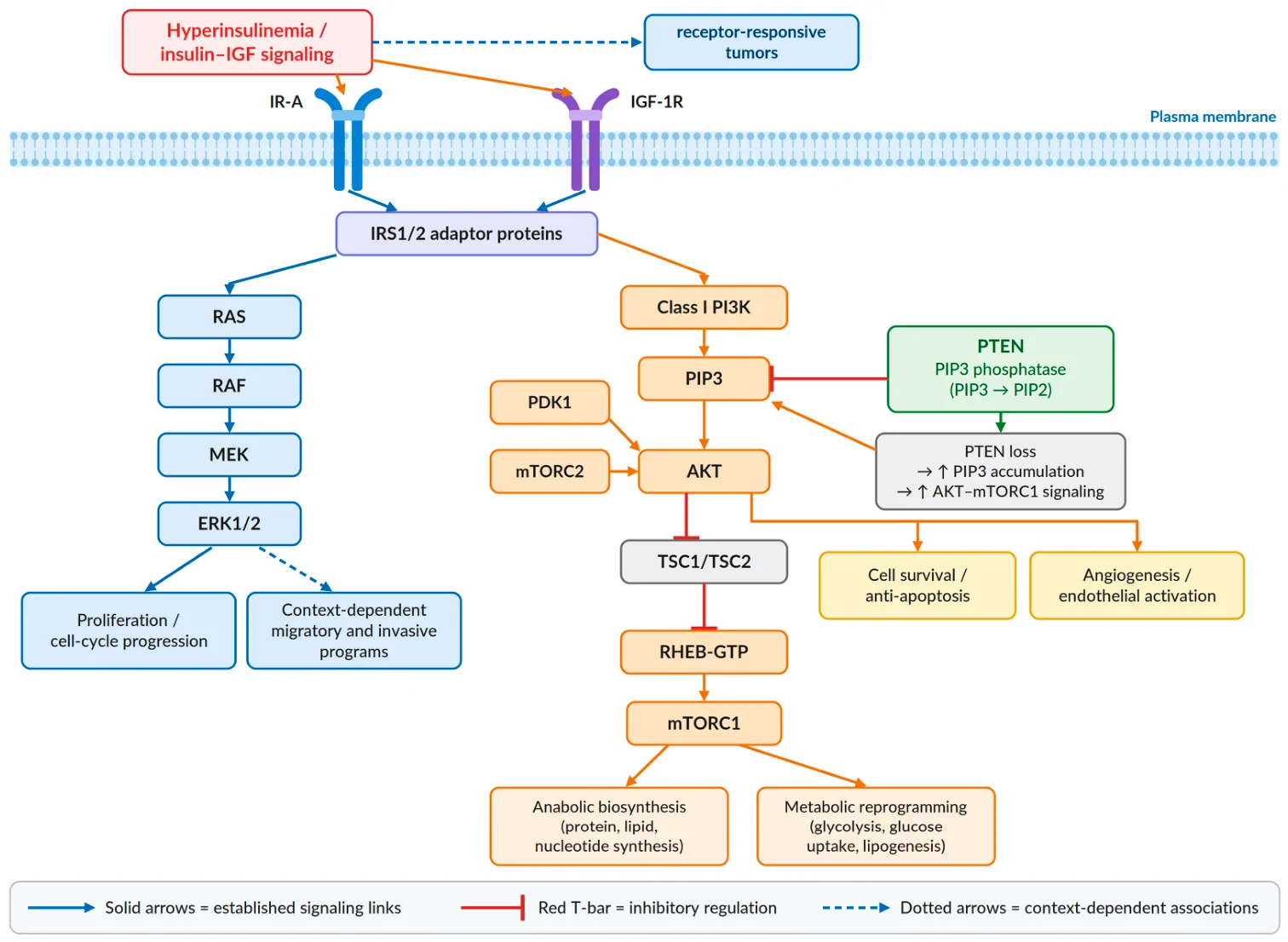
Hyperinsulinemia-Associated Insulin/IGF Receptor Signaling and Integration of the PI3K–AKT–mTOR and RAS–MAPK Pathways in Cancer. In receptor-responsive tumor cells, elevated systemic insulin exposure and context-dependent local IGF ligand activity may activate insulin receptor isoform A (IR-A), insulin-like growth factor 1 receptor (IGF-1R), and IR/IGF-1R hybrid receptors. IRS1/2-associated adaptor proteins link these receptors to the phosphoinositide 3-kinase (PI3K)–AKT–mechanistic target of rapamycin (mTOR) and RAS–RAF–MEK–ERK signaling cascades. Class I PI3K generates phosphatidylinositol-3,4,5-trisphosphate (PIP3), which recruits AKT to the plasma membrane for activation by phosphoinositide-dependent kinase 1 (PDK1) and mTOR complex 2 (mTORC2). AKT-mediated inhibition of the TSC1/TSC2 complex increases RAS homolog enriched in brain (RHEB)-GTP and promotes mTOR complex 1 (mTORC1) activation. PTEN counteracts PI3K signaling by dephosphorylating PIP3 to phosphatidylinositol-4,5-bisphosphate (PIP2). These pathways can support glucose uptake and glycolysis, anabolic biosynthesis, cell survival, proliferation, and context-dependent migratory, invasive, and angiogenic programs. Solid arrows indicate established signaling links, whereas dotted arrows indicate indirect or context-dependent associations. The magnitude and biological consequences of these effects depend on receptor composition and abundance, ligand availability, tumor genotype, nutrient and oxygen availability, microenvironmental conditions, and treatment-induced endocrine feedback.

Sustained activation of IR and IGF signaling networks can promote anabolic metabolic reprogramming by enhancing nutrient uptake, glycolytic and biosynthetic flux, and prosurvival signaling. In hyperinsulinemic states, increased insulin exposure and context-dependent IGF ligand activity may further amplify these effects in tumors with relevant receptor expression or downstream oncogenic alterations, thereby supporting growth, stress adaptation, and therapeutic resistance [16,18]. Insulin/IGF signaling can activate the PI3K–AKT–mTOR and RAS–MAPK pathways, thereby increasing glucose uptake, glycolytic flux, and anabolic biosynthesis [16,17,36]. The receptor-responsive insulin/IGF signaling network and its context-dependent metabolic and oncogenic outputs are summarized in Figure 1.

### 3.2. PI3K–AKT–mTOR Pathway Activation

The phosphoinositide 3-kinase (PI3K)–AKT–mechanistic target of rapamycin (mTOR) pathway is a central regulator of cell growth, survival, and metabolic adaptation [19]. In cancer, this signaling network is frequently dysregulated through alterations affecting receptor tyrosine kinases, activating mutations or amplification of PIK3CA, loss or inactivation of PTEN, and changes in other upstream or pathway-intrinsic regulators [37,38,39]. These alterations can uncouple anabolic signaling from normal growth-factor and nutrient constraints, thereby supporting proliferation, survival, and therapeutic resistance.

Upon growth-factor stimulation, class I PI3Ks generate phosphatidylinositol-3,4,5-trisphosphate (PIP3), which recruits AKT to the plasma membrane. AKT is activated by phosphorylation at Thr308 by phosphoinositide-dependent kinase 1 (PDK1) and at Ser473 by mTOR complex 2 (mTORC2). Activated AKT phosphorylates and inhibits TSC2 within the TSC1/TSC2 complex, relieving its GTPase-activating protein activity toward RAShomolog enriched in brain (RHEB) and thereby increasing RHEB-GTP-dependent activation of mTOR complex 1 (mTORC1) [40,41]. The magnitude and biological consequences of this signaling depend on tumor genotype, microenvironmental nutrient and oxygen availability, and treatment-related stress.

The PI3K–AKT–mTOR network promotes the uptake and utilization of nutrients required for anabolic growth. AKT can increase glucose uptake by promoting plasma-membrane localization and, in some settings, expression of glucose transporters such as GLUT1. It can also enhance glycolytic metabolism through regulation of hexokinase 2 (HK2), phosphofructokinase-related enzymes, and other glycolytic effectors [42,43]. In parallel, PI3K–AKT–mTORC1 signaling can promote glucose-carbon flux through the oxidative and non-oxidative branches of the pentose phosphate pathway (PPP), supporting NADPH-dependent redox buffering and ribose-5-phosphate production for nucleotide synthesis [44,45].

The pathway also promotes de novo lipid synthesis through mTORC1-dependent activation of sterol regulatory element-binding proteins (SREBPs) and supports nucleotide biosynthesis through transcriptional and post-translational mechanisms. mTOR functions in two structurally and functionally distinct complexes. mTORC1 integrates growth-factor and nutrient availability with cellular energy and stress status to regulate cap-dependent mRNA translation, ribosome biogenesis, protein synthesis, lipid synthesis, and nucleotide production, largely through effectors such as S6 kinase 1 and 4E-binding protein 1 [46,47]. mTORC1 also suppresses autophagy under nutrient-replete conditions, in part through inhibitory phosphorylation of ULK1 and other components of the ULK1 complex. However, the biological consequences of autophagy induced by mTORC1 inhibition are context-dependent and can either limit tumor growth or support tumor-cell survival during metabolic stress [48]. mTORC2 functions downstream of growth factor–PI3K signaling to promote AKT activation and regulate cellular programs involved in survival, cytoskeletal organization, migration, and metabolic adaptation [47,49]. Thus, mTORC1 and mTORC2 cooperate with other oncogenic pathways to maintain the biosynthetic and stress-adaptive capacity of cancer cells.

The metabolic effects of PI3K–AKT–mTOR activation should not be interpreted as a uniform suppression of mitochondrial oxidative phosphorylation. Although this pathway frequently increases glucose uptake and glycolytic flux, cancer cells may retain or increase mitochondrial function according to lineage, nutrient availability, hypoxia, and therapeutic pressure [3,18]. PI3K–AKT–mTOR signaling therefore supports metabolic plasticity rather than a single invariant metabolic phenotype, enabling tumor cells to balance ATP production, macromolecule synthesis, and redox homeostasis in changing microenvironments [50,51].

Beyond tumor cells, PI3K–AKT–mTOR signaling contributes to vascular and microenvironmental remodeling. In endothelial cells, VEGF-dependent activation of this pathway supports endothelial survival, proliferation, and glycolytic metabolism required for sprouting angiogenesis [52,53]. It can also influence nitric oxide signaling and fatty acid metabolism, linking angiogenic remodeling to the evolving metabolic state of the tumor microenvironment [54,55].

In hyperinsulinemic states, persistent insulin exposure may amplify PI3K–AKT–mTOR signaling in tumors that retain ligand-responsive IR and/or IGF-1R signaling [56,57]. Such amplification may enhance glucose utilization, biosynthetic metabolism, redox adaptation, and survival signaling, particularly in tumors already harboring pathway-activating lesions or receptor overexpression [18]. However, hyperinsulinemia does not itself account for recurrent genomic alterations of the PI3K–AKT–mTOR pathway. Rather, it may reinforce pre-existing tumor-intrinsic pathway activation in a receptor-, genotype-, and tissue-context-dependent manner. Conversely, pharmacologic PI3K inhibition can induce systemic hyperglycemia and compensatory hyperinsulinemia, which may reactivate IR-dependent PI3K–AKT signaling in tumors and attenuate the efficacy of pathway inhibitors [57]. This distinction is important when considering PI3K, AKT, mTORC1/2, or metabolic enzymes as therapeutic targets in metabolically defined patient subsets [58].

### 3.3. RAS–RAF–MEK–ERK Pathway Activation

The RAS–RAF–MEK–ERK cascade transduces growth-factor and, depending on cellular context, stress-related signals to regulate transcriptional programs, cell-cycle progression, differentiation, migration, and survival. Aberrant activation of this pathway is common in cancer and can arise through receptor tyrosine kinase signaling or tumor-intrinsic alterations in RAS, RAF, or other pathway components [59,60,61].

Following ligand-dependent receptor activation, adaptor proteins such as Shc and IRS family members can couple IR and IGF-1R signaling to RAS activation and downstream RAF–MEK–ERK signaling [16,62]. In tumors expressing IR, IGF-1R, or IR/IGF-1R hybrid receptors, insulin and IGF ligands can therefore link metabolic and growth-factor cues to proliferative and survival programs. In experimental models of diabetes-associated colorectal cancer, insulin and IGF-1 increased ERK1/2 and JNK phosphorylation, promoted cell-cycle progression, and accelerated tumor growth, illustrating a context-specific mechanism through which elevated insulin/IGF signaling may amplify MAPK activity [63]. These findings should not be generalized to all tumor types, because the contribution of RAS–RAF–MEK–ERK signaling to insulin-associated tumor phenotypes depends on receptor composition, oncogenic genotype, and metabolic context.

### 3.4. Coordinated Activation and Crosstalk Between PI3K–AKT–mTOR and RAS–RAF–MEK–ERK Signaling

Insulin and IGF ligands can activate PI3K–AKT–mTOR and RAS–RAF–MEK–ERK signaling through IR, IGF-1R, IR/IGF-1R hybrid receptors, and associated adaptor proteins. In tumors with receptor overexpression or downstream oncogenic alterations, this common upstream input can reinforce parallel engagement of both pathways [62,64]. PI3K–AKT–mTOR signaling is a major regulator of glucose uptake, anabolic metabolism, protein and lipid synthesis, and cell survival, whereas RAS–RAF–MEK–ERK signaling regulates proliferative, differentiation, migratory, and invasive programs; however, their functional outputs overlap substantially [60,65]. Coordinated activation can therefore amplify mitogenic and metabolic signaling, promote survival, and increase the capacity of tumor cells to adapt to therapeutic stress [38,64].

Crosstalk between these cascades arises through shared receptor and adaptor inputs, reciprocal feedback regulation, and convergence on common transcriptional and metabolic effectors, including MYC, HIF-1α, and mTORC1-associated anabolic programs [18]. Through these interactions, tumor cells can integrate growth-factor signaling with nutrient availability, hypoxia, and oncogenic alterations to support metabolic plasticity and, in selected contexts, epithelial–mesenchymal transition, invasion, and therapy adaptation [18,22]. Hyperinsulinemia may amplify signaling through these networks in susceptible tumors but does not itself account for tumor-intrinsic alterations in RAS or PI3K-pathway components [38].

Reciprocal feedback and compensatory signaling can limit the durability of single-node pathway inhibition. Pharmacologic PI3K-pathway inhibition can impair insulin signaling in peripheral tissues, causing systemic hyperglycemia and compensatory hyperinsulinemia. In experimental tumor models, this endocrine feedback can reactivate IR-dependent PI3K signaling and attenuate the antitumor activity of PI3K inhibitors [57]. Inhibition of one signaling cascade can also relieve inhibitory feedback or increase dependence on an alternative signaling route, providing a rationale for molecularly guided combination approaches. However, the therapeutic index of combined PI3K–AKT–mTOR and RAS–RAF–MEK–ERK pathway inhibition may be limited by systemic toxicity, and the value of this strategy is expected to depend on tumor genotype, receptor status, metabolic phenotype, and treatment context [38,66].

Collectively, systemic metabolic dysfunction, hyperinsulinemia, insulin/IGF receptor signaling, tumor-intrinsic oncogenic alterations, and microenvironmental stress responses form an interconnected network that may support cancer progression and metastatic competence. The principal signaling nodes, associated metabolic consequences, and potential contributions to tumor progression and metastasis are summarized in Table 1.

### 3.5. Synergistic Interactions Between Oncogenic Alterations and Insulin Signaling: PTEN Loss and KRAS Activation

#### 3.5.1. PTEN Loss and Hyperactivation of PI3K–AKT–mTOR Signaling

Phosphatase and tensin homolog (PTEN) is a lipid phosphatase that antagonizes PI3K signaling by converting phosphatidylinositol-3,4,5-trisphosphate (PIP3) to phosphatidylinositol-4,5-bisphosphate (PIP2), thereby limiting AKT activation [39,98]. PTEN loss or downregulation is common in many cancers and can promote hyperactivation of PI3K–AKT–mTOR signaling, thereby supporting proliferation, survival, and metabolic remodeling [99]. In several tumor models, PTEN deficiency can increase glucose uptake and glycolytic flux through PI3K–AKT-dependent mechanisms, including enhanced plasma-membrane localization of GLUT1 [69,100]. However, associated changes in mitochondrial metabolism, redox homeostasis, and metabolic dependency are tumor- and context-dependent.

In tumor cells, PTEN loss promotes PIP3 accumulation and facilitates AKT recruitment and activation at the plasma membrane, with consequent activation of mTORC1 [39]. Although crosstalk with RAS–MAPK signaling can occur, it should not be considered a universal direct consequence of PTEN deficiency. In selected experimental systems, residual ligand-responsive signaling may permit insulin or other growth factors to further augment PI3K–AKT activity in PTEN-deficient cells; however, the extent of this effect depends on baseline pathway activation, receptor expression, and feedback regulation [70,99].

Thus, elevated insulin exposure may reinforce PI3K–AKT–mTOR signaling in tumors with PTEN loss, favoring anabolic metabolic programs associated with glycolytic reprogramming and stress adaptation. Whether this interaction creates a clinically meaningful metabolic vulnerability remains to be established across tumor types and molecular subgroups.

#### 3.5.2. Oncogenic KRAS and Insulin Signaling

Oncogenic KRAS is a major driver of initiation, progression, metabolic reprogramming, and therapeutic resistance in several cancer types and can engage PI3K and RAF–MEK–ERK signaling networks [101,102,103]. Hyperinsulinemia may provide a permissive systemic environment that augments ligand-dependent signaling and metabolic outputs in susceptible tumors harboring KRAS activation, PTEN loss, or related pathway lesions [8,56]. Experimental evidence in selected KRAS-mutant pancreatic epithelial models suggests that insulin can enhance PI3K/AKT and ERK1/2 signaling and promote invasive behavior; however, the generalizability of this interaction across KRAS-driven cancers remains unresolved [104].

The magnitude of insulin-associated signaling in KRAS- or PTEN-altered tumors is shaped by tumor lineage, IR/IGF receptor abundance and isoform composition, ligand availability, co-occurring genomic alterations, and microenvironmental metabolic conditions; these variables support metabolically informed, molecularly guided combination strategies rather than a uniform assumption that insulin/IGF-axis targeting will benefit all such tumors [101,103].

## 4. Hyperinsulinemia-Associated Metabolic Reprogramming in Cancer

### 4.1. Glycolysis and the Warburg Effect

Cancer cells reprogram glucose, lipid, and amino acid utilization to support ATP production, redox homeostasis, and the generation of biosynthetic precursors required for proliferation and stress adaptation [1,2]. Many tumors increase glucose uptake through enhanced expression or plasma-membrane localization of glucose transporters, particularly GLUT1, together with increased expression or activity of glycolytic enzymes such as hexokinase 2 (HK2) and lactate dehydrogenase A (LDHA) [105,106].

The Warburg effect describes increased glucose uptake and lactate production despite oxygen availability. This phenotype can support rapid glycolytic flux, carbon diversion into biosynthetic pathways, and redox adaptation; however, mitochondrial oxidative metabolism often remains functional and is remodeled rather than uniformly suppressed [5]. Lactate accumulation and extracellular acidosis can promote tumor progression by reshaping cancer-cell metabolism and by influencing stromal, endothelial, and immune-cell populations within the tumor microenvironment, with consequences for angiogenesis, invasion, and immune suppression [34,35,72]. In selected cancers, elevated intratumoral lactate concentrations, often accompanied by extracellular acidification, have been associated with metastatic behavior, recurrence, and poorer survival [73,74,75].

In tumors with functional IR/IGF receptor signaling, insulin and IGF ligands can activate PI3K–AKT–mTOR signaling, thereby increasing glucose uptake, glycolytic flux, and, in some contexts, PPP activity [16,18]. PI3K–AKT–mTOR activation can regulate glucose transporters and glycolytic effectors, including HK2, PFKFB3 and PFKFB4, PKM2, and LDHA, through direct and indirect mechanisms involving mTORC1, HIF-1α, MYC, and other transcriptional regulators [106]. It can also promote glucose-carbon flux into the PPP and other redox-supporting biosynthetic pathways while variably remodeling mitochondrial substrate use, tricarboxylic acid cycle activity, and oxidative phosphorylation in a tumor- and context-dependent manner [18].

Hyperinsulinemia may amplify these effects in tumors with relevant receptor expression or downstream pathway alterations. PI3K–AKT–mTORC1 activation can increase HIF-1α abundance and cooperate with hypoxic signaling to support glucose uptake, glycolysis, and, in some contexts, PPP flux, contributing to NADPH generation and nucleotide synthesis [18,107]. Collectively, these mechanisms provide a framework through which hyperinsulinemia may reinforce glycolytic and anabolic metabolic programs while contributing to lactate-rich tumor microenvironments.

**Figure 2:**
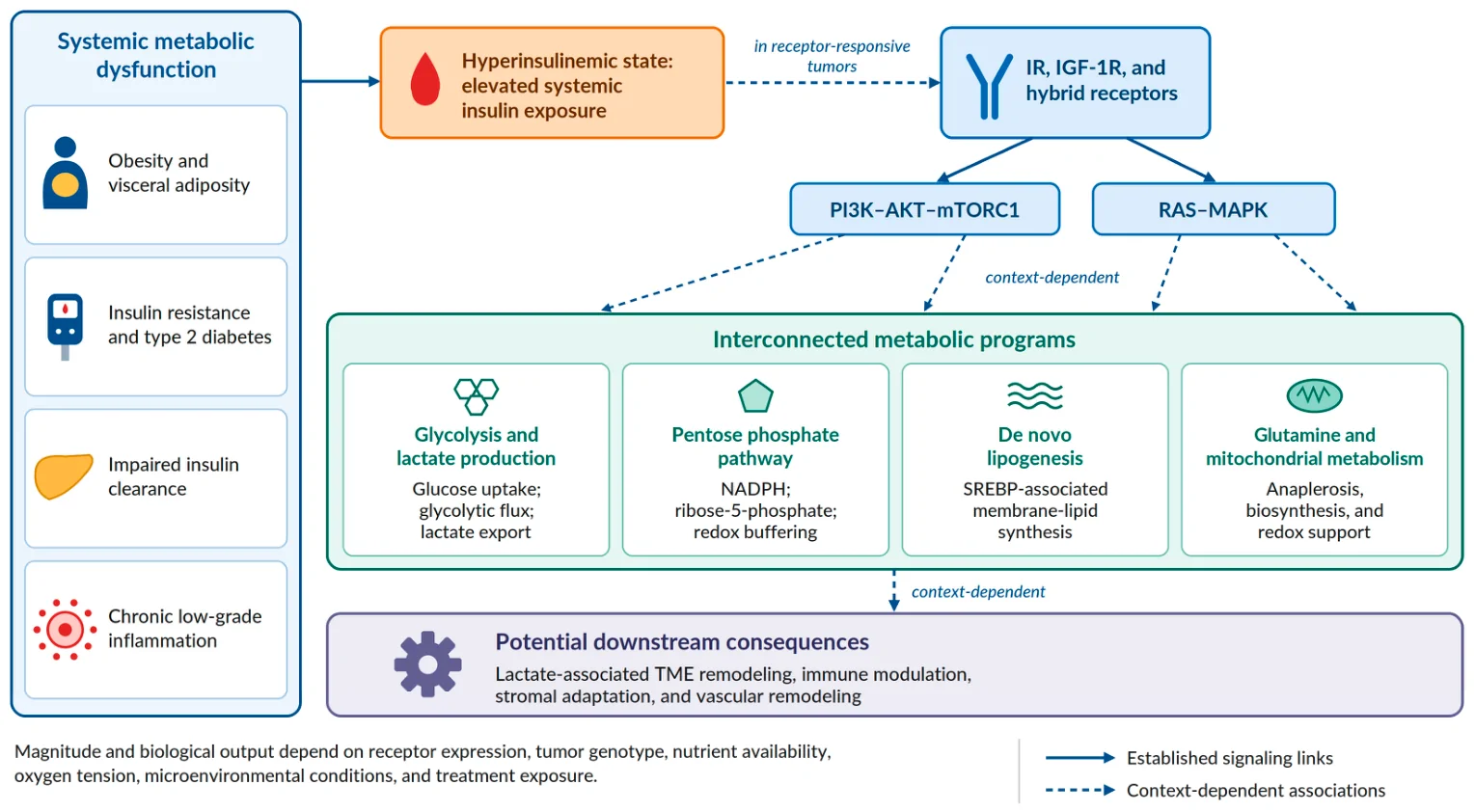
Hyperinsulinemia-Associated Insulin/IGF Signaling and Metabolic Reprogramming in Cancer. Systemic metabolic dysfunction associated with obesity and visceral adiposity, insulin resistance and type 2 diabetes, impaired insulin clearance, and chronic low-grade inflammation can contribute to a hyperinsulinemic state. In receptor-responsive tumors, elevated insulin exposure may engage insulin receptor (IR) isoforms, insulin-like growth factor 1 receptor (IGF-1R), and IR/IGF-1R hybrid receptors, with downstream activation of PI3K–AKT–mTORC1 and RAS–MAPK signaling pathways. These signaling networks can influence interconnected metabolic programmes, including glycolysis and lactate production, pentose phosphate pathway activity, de novo lipogenesis, and glutamine-dependent mitochondrial metabolism. Potential downstream consequences include lactate-associated tumor-microenvironmental remodeling, immune modulation, stromal adaptation, and vascular remodeling. The magnitude and biological consequences of these effects depend on receptor expression, tumor genotype, nutrient availability, oxygen tension, microenvironmental conditions, and treatment exposure. Solid arrows indicate established signaling links, whereas dotted arrows indicate context-dependent associations.

Figure 2 summarizes the interconnected effects of hyperinsulinemia-associated insulin/IGF signaling on glycolysis, PPP activity, de novo lipogenesis, glutaminolysis, redox adaptation, and lactate-associated tumor microenvironmental remodeling in receptor-responsive tumor cells.

### 4.2. Lipid Metabolism and De Novo Lipogenesis

Lipid metabolic reprogramming supports membrane biogenesis, lipid-mediated signaling, redox control, and stress adaptation in cancer [77]. Cancer cells can enhance de novo fatty acid synthesis through increased expression or activity of ATP-citrate lyase (ACLY), acetyl-CoA carboxylase (ACC), fatty acid synthase (FASN), and related enzymes [78]. ACLY generates cytosolic acetyl-CoA from citrate, ACC converts acetyl-CoA to malonyl-CoA, and FASN uses an acetyl-CoA primer, malonyl-CoA, and NADPH to synthesize palmitate [77,78]. The relative contributions of fatty acid oxidation, lipid uptake, and lipid storage vary across tumor types and microenvironmental conditions.

Hypoxia and high glycolytic flux can alter citrate metabolism, redox balance, and lipid-acquisition strategies, thereby modifying the balance among lipid synthesis, storage, uptake, and oxidation in a context-dependent manner. When oxidative glucose metabolism is constrained, cancer cells may generate citrate through reductive carboxylation of glutamine-derived α-ketoglutarate or increase their reliance on extracellular acetate, citrate, or lipids to sustain membrane-lipid synthesis [79]. Cytosolic citrate supplies acetyl-CoA for de novo fatty acid synthesis, whereas malonyl-CoA serves as a substrate for fatty acid synthesis and elongation and can restrain mitochondrial fatty acid oxidation through inhibition of carnitine palmitoyltransferase 1 (CPT1), although the net contribution of fatty acid oxidation varies across tumor contexts [78,79]. These changes can support membrane production, metabolic plasticity, and adaptation to therapeutic stress [79].

In tumors that retain insulin/IGF-responsive receptor signaling, persistent hyperinsulinemia may enhance PI3K–AKT–mTORC1 activity, thereby promoting SREBP-dependent de novo lipogenesis [16,80]. mTORC1-dependent SREBP activation can increase expression of ACLY, ACC, FASN, and stearoyl-CoA desaturase 1 (SCD1), supporting de novo fatty acid and membrane-lipid synthesis required for proliferative growth [81]. Sustained PI3K–AKT–mTOR signaling may also favor lipid-droplet formation and lipid storage in selected tumor contexts, potentially buffering lipotoxic and oxidative stress and providing substrates during nutrient limitation or reoxygenation [77,79].

The oxidative PPP supplies NADPH for antioxidant defense and reductive biosynthesis, whereas the non-oxidative branch exchanges glycolytic intermediates and pentose phosphates to accommodate biosynthetic demand. By supplying NADPH, PPP activity directly links glucose metabolism to fatty acid synthesis and redox homeostasis [45]. Insulin/IGF-associated PI3K–AKT–mTOR signaling may increase glucose-carbon flux toward PPP-associated and lipogenic pathways, although the magnitude of this response remains tumor- and context-dependent [18,45,107]. Collectively, coordinated glycolysis, PPP activity, and SREBP-dependent lipogenesis can supply biosynthetic precursors and reducing equivalents that support cancer-cell proliferation and stress adaptation [45,80].

### 4.3. Glutaminolysis and TCA Cycle Support

Glutaminolysis is an important complementary pathway in cancer metabolism. It provides α-ketoglutarate to replenish tricarboxylic acid (TCA) cycle intermediates and can support mitochondrial metabolism when glucose-derived carbon is constrained. Glutamine also supplies nitrogen for nucleotide and amino acid synthesis and provides glutamate for glutathione production, thereby linking anaplerosis to redox control [82]. Ammonia generated during glutaminolysis, which exists predominantly as ammonium under physiological conditions, can influence intracellular and lysosomal pH homeostasis and modulate autophagy; however, its effects on cell survival are concentration-, cell type-, and context-dependent [108].

Many tumors exhibit increased glutamine uptake and glutaminase activity, but the resulting dependence on glutamine for anaplerosis, biosynthesis, and antioxidant defense varies by tumor lineage, oncogenic genotype, and nutrient environment [82]. mTORC1 can enhance glutamine anaplerosis by promoting CREB2 destabilization and thereby repressing SIRT4 transcription. Because SIRT4 inhibits glutamate dehydrogenase (GDH), this mechanism increases GDH activity and promotes glutamate-to-α-ketoglutarate conversion [83]. Conversely, glutamine availability can promote mTORC1 activation through amino acid-sensing mechanisms that are distinct from those used by leucine, illustrating bidirectional coupling between nutrient metabolism and growth signaling [84]. In ovarian cancer models, inhibition of glutaminolysis enhanced sensitivity to PI3K/mTOR inhibition, supporting glutamine metabolism as a context-dependent determinant of therapeutic response [85].

In tumors with functional IR/IGF receptor signaling, hyperinsulinemia may amplify mTORC1 activity and thereby influence glutamine utilization through downstream regulators such as MYC and the SIRT4–GDH axis [18,83]. However, direct evidence that hyperinsulinemia uniformly increases glutamine-transporter or glutaminase expression across tumor types remains limited. Together, these findings nominate glutaminolysis and mTORC1–glutamine metabolic crosstalk as candidate vulnerabilities for evaluation in molecularly and metabolically defined hyperinsulinemic tumor contexts [82,85].

### 4.4. Pentose Phosphate Pathway and Redox Control

Cancer cells adapt to hypoxia and oxidative or electrophilic stress through metabolic rewiring and stress-response programs, including context-dependent changes in PPP activity, HIF-associated metabolic adaptation, and NRF2-dependent redox buffering. The oxidative PPP generates NADPH and pentose phosphates, whereas the non-oxidative branch reversibly interconverts glycolytic intermediates with ribose-5-phosphate and other pentose phosphates to meet biosynthetic demand. NADPH supports glutathione- and thioredoxin-dependent antioxidant systems and reductive biosynthesis, including fatty acid synthesis; thus, PPP flux can couple redox homeostasis to proliferative capacity in cancer cells [20,45].

Oncogenic signaling and NRF2 activation, often sustained by oxidative stress or disruption of KEAP1-mediated control, can increase the expression of PPP enzymes, including glucose-6-phosphate dehydrogenase (G6PD), 6-phosphogluconate dehydrogenase (PGD), transketolase (TKT), and transaldolase 1 (TALDO1), thereby supporting NADPH generation and nucleotide synthesis [20,21]. Hypoxia can further reprogram glucose metabolism and thereby influence PPP flux; however, the relative engagement of oxidative and non-oxidative PPP branches is tumor- and context-dependent [45,71].

In tumors retaining ligand-responsive IR and/or IGF-1R signaling, hyperinsulinemia may enhance PI3K–AKT–mTOR signaling, a pathway that can support glycolytic metabolism, anabolic growth, and PPP-associated redox and biosynthetic programs [16,18]. In acute myeloid leukemia, persistently high mTORC1 activity promotes glycolytic glucose dependence and creates a vulnerability to G6PD inhibition. In contrast, mTORC1 attenuation can permit glucose-independent survival accompanied by increased mitochondrial oxidative phosphorylation, illustrating a potential metabolic escape route rather than a uniformly favorable therapeutic response [109]. However, direct evidence that insulin exposure consistently increases G6PD expression or PPP flux across diverse tumor types remains limited. Collectively, these observations provide a plausible mechanistic link between systemic hyperinsulinemia and tumor-intrinsic redox adaptation while highlighting context-specific metabolic vulnerabilities that warrant evaluation in defined cancer settings. The therapeutic exploitation of PPP dependence will nevertheless require selective and tolerable approaches, because NADPH-generating pathways are also essential for normal cellular redox homeostasis.

## 5. Lactate-Associated Remodeling of the Tumor Microenvironment in Hyperinsulinemic States

### 5.1. Tumor-Derived Lactate and Stromal Reprogramming in the TME

The tumor microenvironment (TME) is a dynamic ecosystem composed of malignant cells, cancer-associated fibroblasts (CAFs), adipocytes, immune cells, endothelial cells, perivascular cells, and noncellular extracellular-matrix components. Reciprocal interactions among these components generate metabolic heterogeneity and influence tumor growth, invasion, and therapeutic response [110]. In the setting of systemic hyperinsulinemia, insulin/IGF-associated anabolic signaling may increase glycolytic output and contribute to lactate production in receptor-responsive tumor and stromal cells; however, direct evidence that elevated circulating insulin uniformly establishes a lactate-rich TME across tumor types remains limited [72,106].

In some tumor contexts, CAFs adopt glycolytic programs and export metabolites such as lactate, pyruvate, and ketone bodies that can be used by adjacent oxidative tumor cells. This form of metabolic coupling, often termed the reverse Warburg effect, describes a model in which glycolytic CAFs support tumor metabolism through metabolite transfer. Its occurrence, directionality, and quantitative contribution vary substantially across tumor types and microenvironmental states [111]. Hypoxia commonly promotes glycolytic and lactate-producing programs through HIF-dependent transcriptional responses, whereas glutamine metabolism, lipid metabolism, and mitochondrial function are variably remodeled according to tumor and microenvironmental context [71].

Lactate functions both as a metabolic substrate and as a signaling molecule. MCT4 is commonly associated with lactate export from highly glycolytic cells and can contribute to extracellular acidification through H+-coupled transport, whereas MCT1 often supports lactate uptake by oxidative tumor, stromal, or endothelial cells, depending on transporter expression and local lactate and proton gradients [34]. This transporter-mediated lactate shuttling can coordinate metabolic coupling among tumor, stromal, endothelial, and immune-cell populations, while lactate accumulation and extracellular acidosis may influence stromal activation, angiogenesis, tumor-cell invasion, immune-cell function, and therapeutic response [72].

Lactate accumulation and extracellular acidosis can suppress cytotoxic T-cell and natural killer (NK)-cell function and promote immunosuppressive macrophage states [35,74]. In experimental tumor models, tumor-derived lactic acid can promote macrophage polarization toward immunosuppressive, tumor-supportive states through mechanisms involving HIF-1α-dependent transcriptional programs, including VEGF and arginase 1 expression; however, the relevant mechanisms vary across tumor types and cellular contexts [75]. Abnormal tumor vasculature, vascular compression, and extracellular-matrix remodeling can impair perfusion and exacerbate hypoxia, stabilizing HIF-1α and promoting glycolytic and lactate-producing programs [71]. These conditions can favor metabolically adaptable tumor-cell states and, in selected settings, may facilitate invasion and metastatic dissemination.

**Figure 3:**
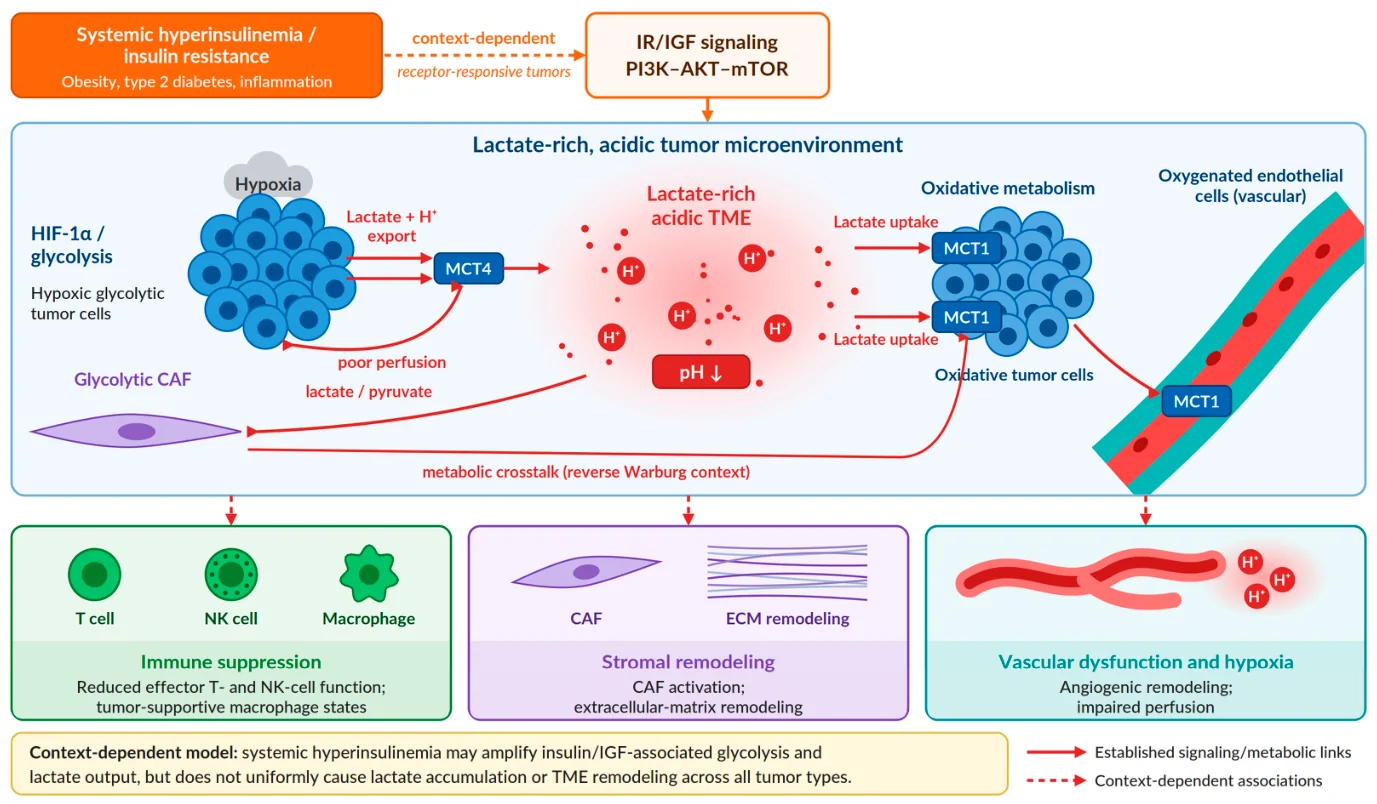
MCT-mediated lactate shuttling and acidosis-associated remodeling of the tumor microenvironment. Hypoxic glycolytic tumor cells can increase HIF-1α-associated glycolytic activity and export lactate together with protons through monocarboxylate transporter 4 (MCT4), contributing to a lactate-rich and acidic tumor microenvironment. Depending on transporter expression and local lactate and proton gradients, monocarboxylate transporter 1 (MCT1) can support lactate uptake by oxidative tumor cells and endothelial cells, thereby facilitating metabolic coupling among distinct cellular populations. Glycolytic cancer-associated fibroblasts (CAFs) may also participate in lactate/pyruvate exchange with tumor cells in selected reverse-Warburg-like contexts. Lactate accumulation and extracellular acidosis can influence immune-cell function, CAF activation and extracellular-matrix remodeling, angiogenic remodeling, and perfusion-associated hypoxia. These effects are context-dependent and vary with tumor lineage, cellular composition, oxygen availability, transporter expression, and local metabolic gradients. Solid arrows indicate established signaling or metabolic transport links, whereas dotted arrows indicate context-dependent associations.

Together, these processes can remodel the TME into a lactate-rich and immunosuppressive ecosystem that may contribute to therapy adaptation. In receptor-responsive tumors, hyperinsulinemia may reinforce this remodeling by increasing insulin/IGF-associated metabolic signaling; however, the causal contribution of systemic insulin excess to TME lactate biology requires direct mechanistic and clinical investigation [35,106]. Figure 3 summarizes the spatial and cellular interactions through which lactate export and uptake, extracellular acidosis, hypoxia, cancer-associated fibroblasts, immune cells, endothelial cells, and vascular dysfunction may contribute to tumor microenvironment remodeling.

### 5.2. Hyperinsulinemia as a Context-Dependent Amplifier of Lactate-Associated TME Remodeling

In tumors retaining ligand-responsive IR and/or IGF-1R signaling, chronic hyperinsulinemia may amplify PI3K–AKT–mTOR activity and support glycolytic and lactate-producing programs. Insulin/IGF-associated signaling and metabolic effects can intersect with hypoxia-responsive pathways, including HIF-1α regulation; however, their magnitude depends on receptor abundance, oncogenic genotype, nutrient availability, oxygen tension, and treatment context [106,107]. Hyperinsulinemia may therefore contribute to, rather than uniformly determine, lactate-rich and acidified microenvironmental states. Conversely, treatment-induced hyperglycemia and compensatory hyperinsulinemia can reactivate IR-dependent PI3K signaling in experimental tumor models exposed to PI3K-pathway inhibitors [57].

Insulin resistance, compensatory hyperinsulinemia, hyperglycemia, and obesity-associated inflammation can create systemic conditions that influence tumor growth and microenvironmental remodeling [13,24]. These states are associated with altered circulating cytokine concentrations and inflammatory signaling within adipose tissue, including IL-6 and TNF-α; however, the relationship between these systemic alterations and local tumor cytokine profiles varies by adipose depot, obesity status, cancer type, and experimental context [67,112].

In hyperinsulinemic-euglycemic clamp studies in rats, lactate infusion reduced insulin-stimulated glycolysis and glucose uptake in skeletal muscle and impaired IRS1/2-associated PI3K and AKT activation [113]. Whether tumor-derived lactate reaches sufficient systemic concentrations to produce a clinically meaningful feedback loop that worsens insulin resistance or hyperinsulinemia in patients remains unresolved. At the tumor-cell level, PI3K–AKT–mTOR signaling can enhance glucose-transporter activity and glycolytic enzyme programs, thereby providing a plausible route through which insulin/IGF signaling could increase lactate output in receptor-responsive tumors [43,114].

Insulin resistance and obesity-associated inflammation may contribute to tumor-supportive microenvironmental features, including immune dysfunction, vascular dysregulation, and metabolic heterogeneity, although their relative contributions vary across tumor types [13]. Insulin/IGF-associated signaling can promote angiogenic programs, including VEGF-related pathways, whereas obesity-associated inflammation, adipose tissue remodeling, and extracellular-matrix changes may further contribute to vascular dysfunction and hypoxia in tumors [33,52,68].

Together, these observations support a model in which systemic metabolic dysfunction can reinforce tumor metabolic and microenvironmental adaptation. Direct evidence connecting hyperinsulinemia to lactate-dependent TME remodeling, however, remains limited and represents an important priority for future mechanistic and clinical studies.

## 6. Insulin–ROS–NRF2 Axis: Antioxidant Adaptation and Redox Resilience

### 6.1. NRF2 in Cancer Redox Control

Cancer cells within the tumor microenvironment experience oxidative stress, inflammatory signaling, and metabolic fluctuations and therefore depend on adaptive redox systems to balance reactive oxygen species (ROS) production and antioxidant defense. Nuclear factor erythroid 2-related factor 2 (NRF2) is a central regulator of this response [115,116]. Under oxidative or electrophilic stress, modification of KEAP1 sensor cysteines impairs KEAP1–Cul3-mediated ubiquitination and proteasomal degradation of NRF2. Newly synthesized NRF2 is thereby stabilized, accumulates, and translocates to the nucleus, where it induces antioxidant, detoxification, and cytoprotective transcriptional programs [20,115,116]. Although transient NRF2 activation can protect normal cells from oxidative damage, persistent activation in established tumors can support redox adaptation, proliferation, and resistance to anticancer therapy [21,117].

Persistent NRF2 activation occurs in a subset of cancers through genomic or functional disruption of the KEAP1–NFE2L2 regulatory axis, chronic oxidative or electrophilic stress, and, in some settings, oncogenic or autophagy-related signaling alterations [115,116,117]. NRF2 induces genes involved in glutathione synthesis and recycling, including GCLC and GCLM; thioredoxin-dependent antioxidant defense, including TXN and TXNRD1; and xenobiotic detoxification, thereby increasing ROS-buffering capacity and limiting oxidative injury [20,116,117]. NRF2 can also promote the expression of enzymes in both the oxidative and non-oxidative PPP, including G6PD, PGD, TKT, and TALDO1, thereby increasing NADPH-generating flux and supporting the production and interconversion of ribose phosphate precursors required for nucleotide synthesis [21]. These changes couple antioxidant defense to nucleotide synthesis and anabolic growth.

In tumors with functional insulin/IGF signaling, hyperinsulinemia may intersect with NRF2-dependent redox programs through PI3K–AKT–GSK3β signaling [19,116]. Hyperinsulinemia-associated nutrient excess and altered mitochondrial or anabolic metabolism may also modify oxidative stress; however, the direction and magnitude of this effect depend on the cellular and microenvironmental context. In receptor-responsive tumors, insulin/IGF–PI3K–AKT signaling may phosphorylate and inhibit GSK3β, reducing GSK3β-dependent phosphorylation of the NRF2 Neh6 degron and subsequent β-TrCP-mediated ubiquitination. This KEAP1-independent regulatory route can increase NRF2 stability and facilitate its nuclear accumulation [118,119].

However, the prevalence, molecular determinants, and clinical significance of NRF2 activation specifically in hyperinsulinemic tumors remain to be defined. This interaction represents a plausible mechanism through which systemic metabolic dysfunction may reinforce redox resilience and resistance to therapy.

### 6.2. Insulin–PI3K–AKT–mTOR–NRF2 Coupling

Insulin/IGF signaling and NRF2-dependent antioxidant responses may intersect to support redox homeostasis and anabolic biosynthesis in cancer cells exposed to metabolic and oxidative stress [20,21]. Insulin and IGF ligands can increase glucose uptake and activate PI3K–AKT–mTOR signaling [19]. In selected experimental systems, PI3K–AKT–mTOR activation has been associated with increased G6PD expression or activity, PPP-associated NADPH generation, and antioxidant capacity; however, the generality of this response across tumor types remains uncertain [18,45,106]. NRF2 can redirect glucose and glutamine metabolism toward NADPH-generating and anabolic pathways, whereas sustained PI3K–AKT activation may provide a complementary signaling context that reinforces these metabolic programs in selected tumors [21].

Hyperinsulinemia-associated insulin/IGF signaling may alter glycolytic, PPP, and glutamine-associated fluxes in receptor-responsive tumors, with context-dependent effects on ROS generation, NADPH availability, and glutathione-dependent redox buffering [12,19]. As discussed above, PI3K–AKT signaling can stabilize NRF2 through inhibition of the GSK3β–β-TrCP degradation axis, providing a potential molecular bridge between insulin/IGF receptor activation and redox adaptation [118,119]. Oncogenic RAS–RAF–MEK–ERK signaling can also modulate NRF2 activity in selected contexts, further illustrating the convergence of growth-factor signaling and redox control [117].

Constitutive NRF2 activation is linked to metabolic plasticity and therapeutic resistance [116,117]. NRF2 activation, including activation associated with KEAP1 loss, can create a selective dependence on G6PD activity; accordingly, genetic or pharmacologic G6PD inhibition suppressed tumor growth in NRF2-activated models and increased the vulnerability of KEAP1-mutant cells [120]. In small-cell lung cancer models, dual PI3K/mTOR inhibition promoted chaperone-mediated autophagic degradation of G6PD, reduced NADPH and ribose-5-phosphate levels, increased oxidative stress, and enhanced radiosensitivity [121]. Whether chronic hyperinsulinemia directly produces sustained NRF2 activation in patients across cancer types remains unresolved. Therapeutic strategies that exploit NRF2-associated metabolic dependencies, rather than indiscriminately suppressing NRF2, may offer a more selective approach in molecularly defined tumors; however, therapeutic benefit, systemic toxicity, and biomarker-guided patient selection require careful evaluation.

**Figure 4:**
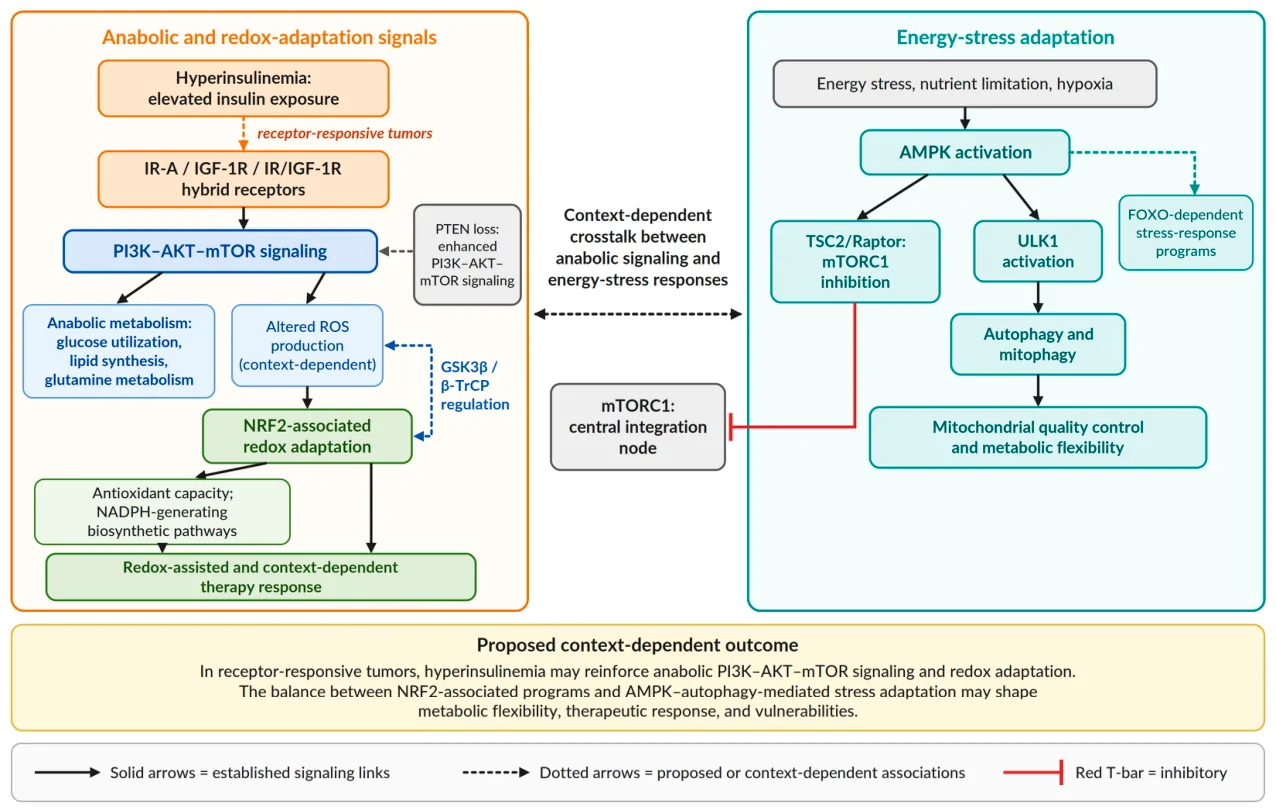
Context-dependent crosstalk between hyperinsulinemia-associated anabolic signaling and energy-stress adaptation in cancer. In receptor-responsive tumors, elevated insulin exposure may activate insulin receptor (IR)-A, insulin-like growth factor 1 receptor (IGF-1R), and IR/IGF-1R hybrid receptor signaling, converging on PI3K–AKT–mTOR pathways. PTEN loss can further enhance PI3K–AKT–mTOR signaling in selected tumors. These inputs may support anabolic metabolism and alter reactive oxygen species (ROS) homeostasis, with potential engagement of NRF2-associated antioxidant and NADPH-generating programs. Conversely, energy stress, nutrient limitation, and hypoxia activate AMP-activated protein kinase (AMPK), which can inhibit mTORC1 through TSC2- and Raptor-dependent mechanisms and activate ULK1, thereby promoting autophagy, mitophagy, mitochondrial quality control, and metabolic flexibility. The balance between anabolic growth signaling and AMPK-mediated stress adaptation is context-dependent and may influence therapeutic response and metabolic vulnerabilities. Solid arrows indicate established signaling links; dotted arrows indicate proposed or context-dependent associations; red T-bars indicate inhibitory interactions.

In parallel with NRF2-mediated redox adaptation, insulin/IGF signaling intersects with the AMPK–mTOR–autophagy axis, discussed in the following section, to reshape cellular responses to metabolic stress. Figure 4 presents an integrative, hypothesis-generating model in which systemic hyperinsulinemia may reinforce PI3K–AKT–mTOR–NRF2-associated anabolic and redox programs in receptor-responsive tumors. Conversely, energy stress, nutrient limitation, and hypoxia may activate AMPK-dependent stress responses, leading to TSC2/Raptor-mediated inhibition of mTORC1 and ULK1-dependent autophagy and mitophagy. The context-dependent crosstalk between these anabolic/redox and AMPK–autophagy-mediated stress-adaptation programs may shape mitochondrial quality control, metabolic flexibility, therapeutic response, and tumour-specific vulnerabilities (Figure 4).

## 7. Integrated Insulin–AMPK–Autophagy Network in Cancer: Metabolic-Stress Adaptation and Therapeutic Vulnerabilities

### 7.1. AMPK–mTOR–Autophagy Axis During Metabolic Stress

AMP-activated protein kinase (AMPK) is a central energy sensor activated during reduced cellular energy charge, typically through LKB1-dependent phosphorylation of the AMPK α-subunit at Thr172 and, in response to elevated intracellular Ca2+, through calcium/calmodulin-dependent protein kinase kinase 2 (CaMKK2)-dependent signaling [86,122]. During metabolic stress, AMPK limits anabolic growth by phosphorylating TSC2 and regulatory-associated protein of mTOR (Raptor), thereby suppressing mTORC1 activity and reducing protein synthesis and other energy-consuming processes [87,88]. AMPK can promote autophagy during energy stress through direct phosphorylation of ULK1 and by relieving mTORC1-mediated inhibition of the ULK1 complex, although the net consequences of autophagy for tumor-cell survival are context-dependent [89,90].

Through these actions, AMPK can support mitochondrial quality control, redox adaptation, and cellular survival during nutrient limitation or oxidative stress. Thus, AMPK may restrain anabolic growth in some settings while promoting stress tolerance and survival in established tumors in others [123]. AMPK also intersects with FOXO-dependent stress-response programs and the PI3K–AKT pathway. Whereas AKT-mediated phosphorylation can inhibit FOXO activity, AMPK-associated stress signaling can support FOXO-dependent adaptive responses; the functional consequences vary with cellular context [91,123,124].

Insulin/IGF signaling activates PI3K–AKT–mTORC1 to promote anabolic metabolism, whereas AMPK often counterbalances these effects under energy stress [88]. PTEN loss and other PI3K-pathway alterations can enhance AKT–mTORC1 signaling and bias cellular signaling toward anabolic growth programs; however, they do not necessarily abolish AMPK-dependent responses to energy stress [70,99]. Thus, AMPK and insulin/IGF–PI3K–AKT signaling converge on mTORC1 to integrate cellular energy status with growth-factor availability.

The relationship between IGF-1R signaling and autophagy is context-dependent. In one hypoxic experimental model, IGF-1R-associated cell survival was linked to enhanced autophagy and reduced ROS accumulation, accompanied by lower canonical PI3K/Akt/mTOR signaling [125]. This finding should be interpreted as a context-specific stress response rather than as a general consequence of IGF-1R activation. It contrasts with the usual autophagy-suppressive role of insulin/IGF–PI3K–AKT–mTORC1 signaling under nutrient-replete conditions and highlights the importance of oxygen availability, nutrient stress, and cell type.

### 7.2. Autophagy, Mitophagy, and AMPK-Dependent Stress Adaptation

Autophagy has context-dependent roles in cancer. By removing damaged organelles and protein aggregates and maintaining proteostasis, basal autophagy can limit ROS accumulation, genomic instability, and malignant transformation during early tumorigenesis. In established tumors, however, autophagy can be co-opted to recycle intracellular substrates and sustain viability during hypoxia, nutrient deprivation, oxidative stress, and therapeutic exposure [92]. The net effect of autophagy depends on tumor genotype, tissue lineage, disease stage, microenvironmental conditions, and treatment context.

ULK1 is a key initiator of autophagy and can contribute to mitophagy, particularly under stress conditions such as hypoxia. In hypoxic breast cancer models, MAPK1/3-dependent phosphorylation of ULK1 promoted BTRC-mediated proteasomal degradation of ULK1, impaired mitophagy, increased mitochondrial ROS and NLRP3 inflammasome activation, and promoted osteolytic bone metastasis [126]. This example underscores why the effects of ULK1 depletion or autophagy inhibition cannot be generalized across cancers: autophagy may support stress adaptation in established tumors, whereas impaired autophagy or mitophagy can also promote malignant progression in specific settings [92,126].

AMPK also has context-dependent actions in cancer. Through phosphorylation of TSC2 and Raptor, AMPK can restrain mTORC1-dependent anabolic signaling and proliferative growth. Under sustained metabolic stress, however, AMPK can promote survival by coordinating catabolic adaptation and autophagy, including direct activation of ULK1. Accordingly, AMPK-dependent autophagy can function either as a therapeutic vulnerability or as a survival mechanism, depending on tumor-specific reliance on autophagic flux, the nature of the metabolic stress, and concurrent treatment [89,91,122]. Both autophagy inhibition and autophagy induction may therefore be beneficial only in molecularly and therapeutically defined settings.

### 7.3. Insulin/IGF–AKT–mTOR Signaling and the AMPK–FOXO–Autophagy Balance

In tumors with functional IR or IGF receptor signaling, chronic hyperinsulinemia may amplify PI3K–AKT–mTOR activity and favor anabolic programs, including protein, lipid, and nucleotide biosynthesis [12,18]. AKT-mediated phosphorylation of FOXO transcription factors promotes 14-3-3 binding, nuclear exclusion, and reduced transcription of stress-responsive and pro-apoptotic genes [124]. Sustained AKT–mTORC1 activity can oppose energy-stress-induced autophagy by maintaining mTORC1-dependent inhibition of ULK1, whereas its effects on AMPK activation itself are context-dependent [89,90].

Under nutrient-replete conditions, mTORC1 suppresses autophagy through inhibitory regulation of the ULK1 complex [92]. In contrast, energy stress activates AMPK, which phosphorylates TSC2 and Raptor to inhibit mTORC1 and directly phosphorylates ULK1 to initiate autophagy [87,90,93]. Persistent insulin/IGF–AKT–mTORC1 signaling may bias cells toward anabolic growth under nutrient-replete conditions; however, the transition to nutrient limitation, hypoxia, or therapeutic stress can induce compensatory catabolic programs and create context-specific metabolic dependencies. When nutrient availability, oxygen tension, or therapeutic exposure becomes unfavorable, sustained anabolic signaling may create selective metabolic liabilities if compensatory autophagy, mitochondrial remodeling, or redox adaptation is insufficient; this possibility requires validation in tumor-specific models [91,92].

AKT signaling also promotes survival through phosphorylation of pro-apoptotic mediators and FOXO transcription factors, thereby reducing the expression of stress-responsive and pro-apoptotic genes in selected contexts [124]. ROS further modulates this network through redox-sensitive regulation of AMPK, mTORC1, and autophagy proteins; its effects on autophagic flux and cell fate depend on ROS intensity, duration, and cellular context [93,94].

PTEN loss or other PI3K-pathway alterations can amplify AKT–mTORC1 signaling and, in tumors retaining residual insulin/IGF receptor responsiveness, may heighten the effects of systemic insulin exposure [18,99]. Hyperinsulinemia may thus favor an anabolism-biased state in receptor-responsive tumors, whereas residual autophagy and metabolic plasticity can permit adaptation to nutrient limitation, hypoxia, and therapy. Strategies that lower systemic insulin exposure, inhibit PI3K–AKT–mTOR signaling, modulate AMPK or autophagy, or restore tumor-suppressive FOXO transcriptional programs may reveal therapeutic vulnerabilities in selected tumors; however, benefits and toxicities are likely to depend on tumor genotype, autophagic dependence, and systemic metabolic state [57,92].

## 8. Therapeutic Strategies for Cancer in Hyperinsulinemic States: Targeting Insulin Signaling, Metabolic Dependencies, and Systemic Metabolic Dysfunction

Therapeutic strategies for cancer arising in hyperinsulinemic or insulin-resistant metabolic contexts can be organized into three complementary approaches: direct inhibition of insulin/IGF receptor signaling and its downstream effectors; targeting of tumor-specific metabolic dependencies; and management of systemic metabolic dysfunction, including treatment-induced hyperglycemia and compensatory insulin feedback. These approaches aim to inhibit tumor-intrinsic growth and survival pathways while addressing host metabolic conditions that may modify treatment response. Because receptor redundancy, metabolic plasticity, and compensatory signaling can limit single-agent activity, rational combinations and biomarker-guided patient selection will be important for clinical evaluation [9,18,95].

Although hyperinsulinemia-associated signaling and metabolic reprogramming provide biologically plausible therapeutic vulnerabilities, clinical translation requires tumor- and patient-specific selection. Relevant considerations include tumor IR/IGF receptor expression and isoform composition, PI3K–AKT–mTOR and RAS–MAPK pathway alterations, metabolic phenotype, baseline glycemic status, treatment-induced hyperglycemia, comorbidities, and anticipated toxicity. Therapeutic strategies, candidate biological contexts, and key translational limitations are summarized in Table 2.

### 8.1. Targeting the Insulin/IGF Axis and PI3K Pathway Feedback

In tumors retaining ligand-responsive IR and/or IGF-1R signaling, hyperinsulinemia may amplify PI3K–AKT–mTOR and RAS–MAPK activity, thereby reinforcing anabolic metabolism, survival signaling, and, in selected contexts, therapy resistance [9,16]. Although IR-A and IGF-1R remain biologically plausible targets, clinical activity of IGF-1R-directed monotherapy has generally been limited outside selected disease settings. Successful therapeutic exploitation will likely require biomarker-guided strategies that account for IR-A and hybrid-receptor signaling, compensatory pathway activation, ligand redundancy, and on-target metabolic toxicity [22,23,134].

Pharmacologic approaches include receptor-blocking antibodies, tyrosine kinase inhibitors, and downstream PI3K, AKT, or mTOR inhibitors. Inhibition of PI3K pathway components, particularly PI3Kα-selective inhibition, can impair insulin signaling in skeletal muscle, adipose tissue, and liver, causing on-target hyperglycemia. The resulting compensatory hyperinsulinemia can reactivate IR-dependent PI3K–AKT signaling in tumor cells and, in preclinical models, attenuate the antitumor activity of PI3K inhibitors [57]. Monitoring and management of PI3K inhibitor-associated hyperglycemia and compensatory insulin feedback are therefore important clinical considerations, particularly in patients with pre-existing insulin resistance or diabetes [95,127].

Preclinical studies indicate that dietary carbohydrate restriction or pharmacologic suppression of insulin feedback can enhance PI3K inhibitor activity in model systems [57]. A stepwise, individualized approach to PI3K inhibitor-associated hyperglycemia—including lifestyle modification, metformin, and selected use of sodium-glucose cotransporter 2 inhibitors or pioglitazone—has been proposed, particularly for alpelisib-treated patients [96]. However, prospective clinical studies are required to establish optimal patient selection, antihyperglycemic strategies, treatment schedules, nutritional feasibility, and long-term safety. Rational combinations with metabolism-directed therapies and interventions designed to reduce compensatory insulin feedback warrant evaluation in molecularly defined settings but should not be broadly extrapolated from preclinical models [57,135].

### 8.2. Targeting Tumor Metabolic Dependencies

In the setting of systemic hyperinsulinemia, receptor-responsive tumors may exhibit enhanced glycolytic and anabolic signaling; however, functional dependence on individual metabolic pathways varies according to tumor lineage, genomic alterations, oxygenation, nutrient availability, microenvironmental interactions, and metabolic plasticity [2,9,18]. In selected tumors, inhibition of GLUT1 or hexokinase activity may restrict glucose entry into glycolysis and the PPP, thereby limiting biosynthetic precursor and NADPH production. Likewise, LDHA inhibition may impair NAD+ regeneration, reduce lactate production, and increase oxidative stress; however, therapeutic activity depends on metabolic compensation, normal-tissue tolerance, and tumor-specific dependence on glycolytic flux [5,105,106].

Lipogenic enzymes, including ACLY, ACC, and FASN, and glutamine-metabolism enzymes such as glutaminase represent candidate metabolic targets in selected tumor contexts. These approaches may exploit lipogenic or glutamine-metabolism dependencies reinforced by insulin/IGF–PI3K–AKT–mTOR signaling, although compensatory nutrient uptake, alternative substrate use, and pathway rewiring can limit therapeutic activity [3,77,82]. The clinical activity of such approaches may also be constrained by tumor heterogeneity, dose-limiting toxicity, and incomplete biomarker definition.

Lactate can accumulate in highly glycolytic tumors and contribute to metabolic symbiosis, extracellular acidification, and immune suppression. In tumors containing spatially distinct glycolytic lactate-exporting and oxidative lactate-consuming compartments, MCT1 inhibition may disrupt lactate uptake and metabolic coupling. In selected preclinical models, this can force oxygenated tumor cells to increase glucose consumption, thereby limiting glucose availability to hypoxic glycolytic cells and increasing their vulnerability [34,128]. However, substrate switching toward glucose, MCT1/MCT4 expression patterns, alternative lactate transport pathways, and the absence of a spatially organized lactate shuttle can limit efficacy; therefore, biomarker-guided patient selection is essential [34,76,128].

### 8.3. Systemic Metabolic Intervention and Biomarker-Guided Metabolic Management

Systemic metabolic intervention represents a complementary component of biomarker-guided treatment for cancer developing or progressing in hyperinsulinemic and insulin-resistant metabolic contexts. Host factors, including fasting insulin and C-peptide as markers of endogenous insulin exposure, HbA1c and fasting glucose as indices of glycemic status, insulin-resistance measures such as HOMA-IR, adiposity, type 2 diabetes, and treatment-induced hyperglycemia, may support exploratory risk stratification in patients in whom systemic metabolic dysfunction could influence tumor signaling or treatment tolerance [9]. These host-level measures should be interpreted alongside tumor-intrinsic characteristics, including IR/IGF-1R expression and receptor isoform composition, PI3K pathway alterations, and metabolic phenotypes such as glycolytic, lipogenic, glutamine-dependent, or lactate-rich states [18,134].

Metformin lowers hepatic glucose output and can reduce circulating insulin in some individuals while potentially influencing tumor-cell energy metabolism through AMPK-dependent and AMPK-independent mechanisms, including context-dependent mTORC1 inhibition [129]. Metformin and other AMPK-modulating agents can also affect FOXO, redox, and autophagy pathways; however, the direction and therapeutic consequences of these effects vary across tumor contexts [97]. Observational studies and meta-analyses have associated metformin use with lower cancer incidence in selected populations; however, these findings are susceptible to confounding by indication, time-related biases, and heterogeneity in diabetes severity and comparator treatments [130]. By contrast, randomized-trial meta-analyses have not demonstrated a consistent reduction in cancer incidence, cancer mortality, or all-cause mortality across tumor types [131]. Metformin is most clearly indicated for glycemic management according to established diabetes care principles. Its role as an anticancer adjunct remains investigational but may warrant evaluation in biomarker-defined patients with insulin resistance, diabetes, obesity, elevated insulin exposure, or treatment-induced hyperinsulinemia. Preclinical studies support evaluation of metformin in rational combinations with insulin/IGF-axis or PI3K–AKT–mTOR pathway inhibitors in metabolically and molecularly selected tumors; however, these strategies require prospective clinical validation [129].

Management of PI3K inhibitor-associated hyperglycemia provides a clinically relevant example of systemic metabolic intervention. Inhibition of PI3Kα-containing complexes impairs insulin signaling in normal metabolic tissues, causing hyperglycemia and compensatory insulin release; this feedback can reactivate tumor IR/IGF-1R–PI3K–AKT signaling and attenuate the antitumor activity of PI3K-pathway inhibitors in preclinical models [57]. Dietary carbohydrate restriction and pharmacologic strategies that suppress insulin feedback enhanced PI3K inhibitor activity in these models [57]. In clinical practice, individualized glucose monitoring and antihyperglycemic management may facilitate continuation of PI3K-targeted therapy; however, whether suppression of insulin feedback consistently improves antitumor efficacy in patients remains to be established [95,96,127]. In analyses from the SOLAR-1 and BYLieve settings, SGLT2 inhibitor use was associated with lower rates of severe hyperglycemia and fewer alpelisib dose modifications; however, these findings require careful interpretation because of nonrandomized comparisons, patient selection, and the potential risks of volume depletion, genitourinary infection, and euglycemic ketoacidosis [127].

Individualized weight management, structured physical activity, dietary counseling, and multidisciplinary endocrine co-management can improve cardiometabolic health and may improve insulin sensitivity, particularly in patients with overweight, obesity, insulin resistance, or type 2 diabetes [132]. These interventions should be adapted to disease stage, treatment-related toxicities, nutritional status, sarcopenia risk, and the presence of involuntary weight loss or cachexia. Their direct effects on tumor control, tumor microenvironment remodeling, angiogenesis, or response to anticancer therapy remain to be established in prospective trials [133]. Accordingly, systemic metabolic intervention should not be considered a uniform anticancer treatment but may complement tumor-directed therapy in metabolically and molecularly selected patients, pending prospective validation of safety, treatment tolerability, and cancer-specific clinical benefit.

## 9. Testable Hypotheses and Experimental Strategies

The association between hyperinsulinemia and cancer does not, by itself, establish direct metabolic effects in tumor cells, identify the mechanisms of stress adaptation, or determine which tumors depend on insulin. Previous reviews have established the biological rationale and clinical relevance of the insulin/IGF axis; the following framework focuses on separating these questions experimentally rather than treating them as interchangeable [9,12,24].

Recent studies reinforce the need to distinguish host-level associations from tumor-cell mechanisms: genetic scores representing insulin resistance-linked hyperinsulinemia were associated with colorectal cancer, whereas scores for insulin resistance without hyperinsulinemia were not associated in the same analysis [136]. A separate UK Biobank analysis linked machine learning-predicted insulin resistance to cancer incidence, but did not test insulin-induced tumor metabolic flux [137]. Such studies strengthen the rationale for mechanistic investigation without establishing the three hypotheses below.

### 9.1. Direct Insulin-Dependent Metabolic Remodeling

Hypothesis: Clinically relevant insulin elevations directly alter selected tumor metabolic fluxes at fixed extracellular glucose. This could be tested in tumor cells and patient-derived organoids using clinically justified and, separately, supraphysiological insulin concentrations. Glucose should be maintained and monitored throughout incubation, with matched cell number, viability, cell-cycle state, and defined ligand conditions. Acute signaling should be assessed separately from sustained changes in glucose uptake, lactate secretion, respiration, and isotope-resolved metabolism; INSR depletion/re-expression and IGF1R controls would test receptor involvement.

At 100 nM insulin, Rabin-Court and colleagues observed increased glucose uptake across six cell lines but no increase in the mitochondrial glucose-oxidation contribution in three [138]. Minn and colleagues found no significant increase in FDG-derived tumor glucose uptake during an acute clamp in six lymphoma patients, without excluding chronic effects [139].

Challenging outcomes: No prespecified, biologically meaningful flux change despite receptor engagement and adequate assay sensitivity, or effects confined to supraphysiological exposure, would challenge the hypothesis or its physiological relevance. Persistence after INSR removal would challenge receptor attribution rather than exclude all direct insulin effects.

### 9.2. Insulin-Dependent Adaptation to Metabolic Stress

Hypothesis: Elevated insulin supports survival during nutrient stress through context-dependent AMPK, NRF2, and autophagy responses. A nutrient availability × insulin design should measure AMPK/NRF2 signaling, survival, recovery after refeeding, redox balance, and autophagic flux, with glucose and amino acid restriction tested separately. Timed AMPK, NFE2L2, and ATG7 or ULK1/2 perturbations with rescue would test causality. An insulin pre-exposure/washout arm could test whether prior exposure establishes NRF2-dependent defense capacity that improves subsequent stress survival.

Mechanistic rationale includes AMPK-dependent NADPH maintenance, NRF2-associated metabolic remodeling, and complementary NRF2/autophagy protection during glucose deprivation [21,91,140]. However, AMPK can restrain ULK1 while preserving later autophagic competence, so AMPK activation does not necessarily indicate increased autophagic flux [141]. Glucose-dependent insulin-associated redox protection provides further rationale, but not proof of AMPK–NRF2–autophagy integration [142].

Challenging outcomes: Absence of a survival or recovery benefit would challenge the protective hypothesis under the tested conditions. Persistence after validated mediator disruption would challenge that mediator’s necessity, while absence of a washout-persistent benefit would challenge the preconditioning extension.

### 9.3. Molecular Predictors of Insulin-Dependent Metabolic Responses

Hypothesis: PTEN, PIK3CA, and IR-A/IR-B modify metabolic insulin dependence, but may be context-dependent rather than universal predictors. Isogenic models should compare PTEN deletion/restoration, PIK3CA alleles or depletion, and receptor-isoform reconstitution at comparable abundance. Dose–response, insulin withdrawal, and rescue experiments should quantify metabolic changes from baseline and test molecular-state × insulin × nutrient interactions.

PIK3CA/PTEN manipulations altered growth-factor dependence and dietary-restriction responses, whereas PTEN loss did not uniformly modify insulin-mediated proliferative responses during PI3K inhibition [57,143]. IR-A-associated metabolic remodeling and isoform-specific transcriptional responses support functional investigation, but do not establish universal predictors [144,145]. Recent breast cancer studies illustrate cell-context-dependent growth and migration and limitations of total-IR measurements without isoform resolution [146,147].

Challenging outcomes: Demonstrated equivalence of metabolic responses and withdrawal sensitivity across molecular states would challenge standalone predictive value. A nonsignificant difference alone would not establish equivalence; nutrient-specific predictive effects would support a conditional biomarker model.

### 9.4. Interpretation and Translational Priorities

These hypotheses distinguish metabolic responsiveness, stress adaptation, and insulin dependence. Negative findings should be interpreted within the tested model and pathway, while clinical translation requires prospective validation of functional markers for patient stratification, treatment selection, and metabolic safety, as discussed in the following section.

## 10. Future Directions: Precision Metabolic Oncology and Multidisciplinary Integration

The mechanistic hypotheses and experimental approaches outlined in the preceding section provide a framework for evaluating when systemic insulin exposure is functionally relevant to tumor metabolism and survival. Translating this framework into clinical strategies will require prospective validation of candidate biomarkers and careful assessment of host metabolic status. Metabolic plasticity, tumor heterogeneity, and adaptive resistance remain major challenges in cancers developing or progressing within hyperinsulinemic and insulin-resistant metabolic states. Rational combinations of insulin/IGF-axis modulators, pathway inhibitors, and metabolism-directed therapies therefore warrant evaluation in biomarker-defined settings alongside standard modalities such as chemotherapy, radiotherapy, and immunotherapy [9,18]. Future trials should prospectively capture baseline and on-treatment insulin exposure, glycemic status, body composition, metabolic comorbidities, treatment-induced hyperglycemia, tumor receptor context, and pathway alterations to distinguish predictive biomarkers from prognostic metabolic correlates.

Dual- or multi-targeted approaches involving IGF-1R, IR, ligand neutralization, or downstream pathway inhibition have a strong mechanistic rationale but require biomarker-guided development, dose and schedule optimization, and rigorous assessment of metabolic toxicity. Clinical translation has been limited by receptor redundancy, hybrid-receptor signaling, compensatory insulin feedback, treatment-associated hyperglycemia, and the absence of validated predictive biomarkers [17,23,134]. Exploratory biomarkers requiring prospective validation include fasting insulin, C-peptide, insulin-resistance indices, glycemic status, circulating IGF ligands and IGF-binding proteins, tumor IR/IGF-1R expression and receptor isoform composition, PI3K pathway alterations, and metabolic imaging or transcriptomic signatures [12]. These measures should be evaluated for their ability to identify treatment-relevant insulin dependence rather than assumed to be predictive on the basis of association alone.

Weight management, dietary modification, and structured physical activity can improve cardiometabolic health and may improve insulin sensitivity, particularly in patients with overweight, obesity, insulin resistance, or type 2 diabetes. However, these interventions should be individualized according to disease stage, treatment-related toxicities, nutritional status, sarcopenia risk, and the presence of involuntary weight loss or cachexia. Their direct effects on tumor control, the tumor immune microenvironment, angiogenesis, or response to systemic anticancer therapy require prospective validation [133].

Cancers occurring in patients with persistent hyperinsulinemia, obesity, type 2 diabetes, or insulin resistance represent clinically meaningful metabolic contexts rather than a uniform biological subgroup. An integrated precision-metabolic approach linking endocrinology, oncology, metabolism, clinical nutrition, and exercise medicine may facilitate safe management of systemic metabolic dysfunction while enabling molecularly guided anticancer therapy [9,12]. Future progress will require alignment of experimentally defined mechanisms with prospectively validated biomarkers and clinically meaningful outcomes, while preserving the distinction between systemic metabolic improvement and demonstrated antitumor benefit.

## Figures and Tables

**Table 1:** Hyperinsulinemia-Associated Signaling and Metabolic Mechanisms Linking Systemic Metabolic Dysfunction to Cancer Progression and Metastasis.

Systemic or Microenvironmental Context	Principal Signaling Nodes	Major Metabolic Consequences	Potential Contributions to Cancer Progression and Metastasis	Representative References
Insulin resistance and compensatory hyperinsulinemia	IR, IR-A, IGF-1R, IR/IGF-1R hybrid receptors, PI3K–AKT–mTOR, RAS–RAF–MEK–ERK	Increased nutrient uptake, glycolytic and anabolic signaling, and prosurvival programs	Enhanced tumor growth and survival; context-dependent support of epithelial–mesenchymal plasticity, invasion, angiogenesis, and metastatic competence	[8,9,12,16,30]
Obesity-associated adipose dysfunction and inflammation	IL-6, TNF-α, adipokines, IR/IGF signaling, MAPK-associated inflammatory pathways	Insulin resistance, altered substrate and lipid availability, oxidative stress, and inflammatory signaling	A tumor-supportive stromal milieu that may facilitate tumor growth, vascular remodeling, invasion, and dissemination	[8,13,24,33,67,68]
Tumor-intrinsic PI3K activation or PTEN loss	PI3K, AKT, mTORC1/2, PTEN, FOXO, SREBP	Increased glucose utilization, glycolysis, PPP flux, protein, lipid, and nucleotide synthesis, and redox buffering	Proliferative fitness, stress tolerance, resistance to cell death, and metabolic flexibility during tumor progression	[18,37,39,65,69,70]
RAS–MAPK activation and insulin/IGF crosstalk	RAS, RAF, MEK, ERK, JNK, PI3K–AKT	Altered glycolysis, anabolic biosynthesis, mitochondrial adaptation, and nutrient utilization	Tumor growth, migration, invasion, and adaptive resistance; potential cooperation with PI3K pathway activation	[16,38,59,60,63]
Hypoxia and oxidative stress	HIF-1, NRF2, KEAP1, PPP, G6PD, AMPK	Glycolytic reprogramming, NADPH generation, antioxidant defense, and redox homeostasis	Survival under hypoxic or oxidative stress, therapy adaptation, and selection of dissemination-competent cell states	[20,21,71]
Lactate accumulation and extracellular acidosis	LDHA, MCT1/MCT4, HIF-1, ERK/STAT3-associated signaling	Lactate shuttling, extracellular acidification, and stromal and immune metabolic reprogramming	Immune suppression, matrix remodeling, angiogenesis, and a microenvironment that may support invasion and metastatic colonization in selected contexts	[34,35,72,73,74,75,76]
Lipid metabolic reprogramming	mTORC1, SREBP1/2, ACLY, ACC, FASN, fatty acid oxidation pathways	De novo lipogenesis, membrane synthesis, lipid storage or oxidation, and redox buffering	Enhanced membrane and energy supply, oxidative-stress tolerance, and survival during dissemination and colonization	[77,78,79,80,81]
Glutamine and mitochondrial adaptation	GLS, mTORC1, SIRT4, TCA-cycle anaplerosis, autophagy	Carbon and nitrogen supply, mitochondrial metabolism, nucleotide synthesis, and redox support	Metabolic flexibility under nutrient limitation and potential resistance to targeted therapies	[50,82,83,84,85]
AMPK–mTOR–autophagy stress response	LKB1, AMPK, TSC2, Raptor, mTORC1, ULK1, ROS	Energy conservation, autophagic recycling, mitochondrial quality control, and NADPH homeostasis	Context-dependent survival during nutrient deprivation, detachment, oxidative stress, and therapeutic exposure	[86,87,88,89,90,91,92,93,94]
Therapy-induced metabolic feedback	PI3K inhibition, hyperglycemia, compensatory insulin secretion, IR/IGF-1R feedback	Potential restoration of downstream signaling and anabolic metabolic programs through compensatory insulin feedback	Reduced depth and durability of PI3K pathway inhibition; rationale for selected insulin-feedback-lowering combination strategies	[57,95,96,97]

Note: Systemic metabolic dysfunction, including obesity-associated insulin resistance, compensatory hyperinsulinemia, hyperglycemia, and chronic low-grade inflammation, can interact with tumor-intrinsic genetic alterations and microenvironmental stress signals. Activation of IR, IGF-1R, and IR/IGF-1R hybrid receptors can engage the PI3K–AKT–mTOR and RAS–RAF–MEK–ERK pathways, with downstream effects on nutrient uptake, glycolysis, anabolic biosynthesis, redox adaptation, survival, and context-dependent invasive or metastatic phenotypes. Hypoxia, lactate accumulation, extracellular acidosis, stromal remodeling, and immune-cell metabolic reprogramming may further modify these outcomes. The listed relationships vary according to tumor lineage, receptor expression, genomic alterations, microenvironmental conditions, disease stage, and therapeutic exposure. Representative references are not exhaustive, and the table does not imply that all pathways are active in every tumor type or establish causality in humans. Abbreviations: ACC, acetyl-CoA carboxylase; ACLY, ATP-citrate lyase; AKT, protein kinase B; AMPK, AMP-activated protein kinase; ERK, extracellular signal-regulated kinase; FASN, fatty acid synthase; FOXO, forkhead box O; G6PD, glucose-6-phosphate dehydrogenase; GLS, glutaminase; HIF-1, hypoxia-inducible factor 1; IGF-1R, insulin-like growth factor 1 receptor; IL-6, interleukin-6; IR, insulin receptor; IR-A, insulin receptor isoform A; JNK, c-Jun N-terminal kinase; KEAP1, Kelch-like ECH-associated protein 1; LDHA, lactate dehydrogenase A; LKB1, liver kinase B1; MAPK, mitogen-activated protein kinase; MCT, monocarboxylate transporter; mTOR, mechanistic target of rapamycin; NADPH, nicotinamide adenine dinucleotide phosphate; NRF2, nuclear factor erythroid 2-related factor 2; PI3K, phosphoinositide 3-kinase; PPP, pentose phosphate pathway; PTEN, phosphatase and tensin homolog; Raptor, regulatory-associated protein of mTOR; RAS, rat sarcoma oncogene homolog; ROS, reactive oxygen species; SREBP, sterol regulatory element-binding protein; TCA, tricarboxylic acid; TNF-α, tumor necrosis factor-α; TSC2, tuberous sclerosis complex 2.

**Table 2:** Therapeutic Opportunities, Biomarker-Guided Strategies, and Translational Limitations in Hyperinsulinemic Cancer Contexts.

Strategy	Therapeutic Rationale and Candidate Context	Major Limitations and Translational Considerations	Representative References
Insulin/IGF-axis inhibition	Targets IR, IR-A, IGF-1R, or IR/IGF-1R hybrid receptor-dependent PI3K–AKT–mTOR and RAS–MAPK signaling in tumors with evidence of ligand- and receptor-responsive signaling. May be most relevant in tumors with high IR/IGF-1R expression, relevant receptor isoform composition, IGF ligand activity, or an insulin-responsive metabolic phenotype.	Receptor redundancy, IR/IGF-1R hybrid receptors, compensatory pathway activation, and possible metabolic toxicity. Clinical activity of IGF-1R-directed monotherapy has often been limited; biomarker-based patient selection is needed.	[12,16,17,18,22,23,36]
PI3K–AKT–mTOR inhibition with insulin-feedback control	Combines tumor-intrinsic PI3K–AKT–mTOR blockade with strategies intended to prevent or attenuate PI3Kα inhibitor-associated hyperglycemia and compensatory hyperinsulinemia. Candidate settings include PIK3CA-altered or pathway-dependent tumors treated with PI3K pathway inhibitors.	Requires individualized baseline metabolic assessment and glucose monitoring. The choice, timing, and safety of insulin-suppressive or glucose-lowering interventions require prospective evaluation; preclinical efficacy gains should not be equated with proven survival benefit.	[18,57,65,95,96,127]
Glycolysis targeting	Targets glucose uptake or glycolytic flux through GLUT1, HK2, PFKFB3/PFKFB4, PKM2, or LDHA. Candidate tumors include those with high glucose uptake, glycolytic gene expression, hypoxia-associated glycolysis, or high lactate production.	Metabolic plasticity, compensation by oxidative phosphorylation or alternative nutrient pathways, incomplete tumor selectivity, and toxicity to normal glucose-dependent tissues. Biomarker-defined dependency remains necessary.	[5,7,105,106,114]
Lipid and glutamine targeting	Targets ACLY, ACC, FASN, SREBP-associated lipogenesis, selected fatty acid oxidation pathways, glutaminase, or glutamine-dependent anaplerosis. Candidate contexts include lipogenic tumors, glutamine-dependent tumors, and cancers with mTORC1/SREBP activation or RAS-driven metabolic dependencies.	Tumors can compensate through exogenous lipid uptake, alternative carbon sources, macropinocytosis, or pathway rewiring. Dependencies are lineage- and microenvironment-specific, so rational combinations and pharmacodynamic biomarkers are needed.	[77,78,79,80,81,82,83,85]
Lactate-metabolism targeting	Targets lactate generation, uptake, or export through LDHA, MCT1, MCT4, or related lactate-handling pathways. Candidate settings include lactate-rich tumors displaying metabolic symbiosis, extracellular acidosis, or immunosuppressive metabolic microenvironments.	MCT expression is heterogeneous; MCT4-selective targeting remains challenging; tumors may bypass lactate blockade through metabolic compensation. Evidence for a uniquely hyperinsulinemia-dependent lactate vulnerability is limited.	[34,72,74,75,76,128]
Metformin and AMPK-directed metabolic modulation	May lower systemic insulin exposure and influence cellular energy stress, AMPK, mTORC1, redox regulation, and autophagy. Metformin is most clearly relevant for glycemic management in clinically appropriate patients with diabetes or insulin resistance; its role as an anticancer adjunct remains investigational.	Randomized studies and meta-analyses have not established a uniform cancer-outcome benefit. Direct tumor-cell effects cannot be readily separated from systemic metabolic effects; renal function, lactic acidosis risk, drug interactions, and treatment context require clinical consideration.	[91,97,129,130,131]
Lifestyle intervention and integrated metabolic care	Individualized weight management, structured physical activity, dietary counseling, and endocrine co-management can improve insulin sensitivity and cardiometabolic health. This approach is particularly relevant for patients with obesity, diabetes, insulin resistance, or PI3K inhibitor-associated hyperglycemia.	Direct causal effects on tumor response, immune remodeling, metastasis, and survival are not established across cancer types. Feasibility, nutritional status, sarcopenia risk, treatment toxicity, and cancer-specific contraindications should be assessed individually.	[10,13,132,133]
Biomarker-guided combinations	Integrates systemic indices of insulin resistance and glycemic status with tumor IR/IGF receptor expression and isoform composition, PI3K pathway genotype, RAS/MAPK activation, metabolic imaging or transcriptomic signatures, lactate/redox phenotype, and dynamic treatment-induced metabolic feedback.	Validated predictive biomarkers, optimal thresholds, serial monitoring approaches, intervention sequence, and safety of multidrug metabolic combinations require prospective clinical validation.	[12,18,57,65,95,127,134]

Note: This table summarizes therapeutic strategies that may target the interaction among systemic hyperinsulinemia, insulin/insulin-like growth factor (IGF) signaling, oncogenic PI3K–AKT–mTOR and RAS–MAPK pathway activation, and metabolic reprogramming in cancer. Candidate contexts identify biologically plausible settings for investigation rather than treatment indications. Clinical utility is expected to depend on tumor type, genotype, receptor expression, systemic metabolic status, prior therapy, comorbidities, and treatment-related toxicity. Evidence ranges from mechanistic and preclinical studies to clinical management guidance and selected human studies. Representative references are not exhaustive. None of the strategies listed should be interpreted as a universal treatment approach or as evidence of proven survival benefit in hyperinsulinemic patients with cancer. Abbreviations: ACC, acetyl-CoA carboxylase; ACLY, ATP-citrate lyase; AKT, protein kinase B; AMPK, AMP-activated protein kinase; FASN, fatty acid synthase; GLUT1, glucose transporter 1; HK2, hexokinase 2; IGF, insulin-like growth factor; IGF-1R, insulin-like growth factor 1 receptor; IR, insulin receptor; IR-A, insulin receptor isoform A; LDHA, lactate dehydrogenase A; MAPK, mitogen-activated protein kinase; MCT, monocarboxylate transporter; mTOR, mechanistic target of rapamycin; PFKFB, 6-phosphofructo-2-kinase/fructose-2,6-bisphosphatase; PI3K, phosphoinositide 3-kinase; PIK3CA, phosphatidylinositol-4,5-bisphosphate 3-kinase catalytic subunit alpha; PKM2, pyruvate kinase M2; RAS, rat sarcoma oncogene homolog; SREBP, sterol regulatory element-binding protein.

## Data Availability

No new data were created or analyzed in this study. Data sharing is not applicable to this article.
